# Participants’ experiences of potential adverse effects of an intervention to improve critical thinking about health choices: a qualitative cross-trial process evaluation in Kenya, Rwanda and Uganda

**DOI:** 10.1136/bmjopen-2025-104236

**Published:** 2025-10-21

**Authors:** Matt Oxman, Faith Chesire, Michael Mugisha, Ronald Ssenyonga, Allen Nsangi, Andrew D Oxman, Atle Fretheim, Sarah Rosenbaum, Margaret Kaseje, Nelson Sewankambo, Monica Melby-Lervåg, Simon Lewin

**Affiliations:** 1 Centre for Epidemic Interventions Research, Norwegian Institute of Public Health, Oslo, Norway; 2 Oslo Metropolitan University Faculty of Health Sciences, Oslo, Norway; 3 Tropical Institute of Community Health and Development, Kisumu, Kenya; 4 Institute of Health and Society, University of Oslo, Oslo, Norway; 5 School of Public Health, University of Rwanda, Kigali, Rwanda; 6 Makerere University College of Health Sciences, Kampala, Uganda; 7 Department of Public Health, Muni University, Arua, Uganda; 8 Centre for Research on Special Needs Education and Inclusion, University of Oslo Faculty of Educational Sciences, Oslo, Norway; 9 Health Systems Research Unit, South African Medical Research Council, Cape Town, South Africa; 10 Norwegian University of Science and Technology Faculty of Medicine and Health Sciences, Ålesund, Norway

**Keywords:** PUBLIC HEALTH, Health Education, Health Literacy, QUALITATIVE RESEARCH

## Abstract

**Abstract:**

**Objectives:**

To explore participants’ experiences of potential adverse effects of the Informed Health Choices secondary school intervention across three trial sites and to revise a framework of potential adverse effects of interventions to improve critical thinking about health choices.

**Design:**

This was a qualitative study. We extracted and analysed relevant data from separate process evaluations in each country. Data came from surveying teachers, observing lessons and group and individual interviews with students, teachers and other stakeholders. We modified and applied framework analysis, including five stages: (1) development of an initial framework of potential adverse effects, (2) familiarisation with the data, (3) indexing, (4) abstraction and synthesis and (5) revising the framework. We applied reflexive strategies individually and as a team.

**Setting:**

Lower secondary school in five randomly sampled subcounties of Kisumu County in Kenya, districts representing all five provinces in Rwanda, and six districts in the central region of Uganda, between 2022 and 2024.

**Participants:**

Students and teachers in the intervention arms of the trials, parents of students in the intervention arms and administrators at intervention schools, as well as curriculum developers and policy-makers.

**Intervention:**

The intervention involved providing teachers with a 2–3-day training workshop, and digital classroom resources, including lesson plans for 10 lessons to be delivered over the course of one semester.

**Results:**

We generated findings about potential increases in adverse misunderstandings, anxiety related to transfer of learning, adversely experienced cognitive dissonance, work or schoolwork-related stress, inequity, conflicts and waste. The revised framework includes the same categories of potential adverse effects as our initial framework: decision-making harms, psychological harms, equity harms, group and social harms, waste and other harms. We revised other elements of the framework, including definitions of the categories and its structure.

**Conclusions:**

This study provides insight into the potential adverse effects of interventions to improve critical thinking about health choices. The findings complement those of the trials and country-level process evaluations.

**Strengths and limitations of this study**

- We explored the experiences of a variety of interest-holders, including students, teachers, parents and policy-makers.
- We analysed data collected using a variety of methods, including survey (open-ended items), observation and individual and group interviews.
- To collect and analyse the data, we used an iteratively developed framework.
- We did not explore the importance that interest-holders place on different effects, nor did we explore differences across different contexts or populations.

## Introduction

 Both qualitative and quantitative methodological purists might baulk at the use of ‘effects’ in the title of this report, as well as other quantitative terminology in the main text. An ‘effect’ of an intervention is an increase or decrease in an outcome caused by the intervention. In most cases, randomised trials are required to be certain of the effects of most interventions, because intervention effects are rarely dramatic, and randomisation is the only method of controlling for unmeasured or unknown confounding.^1 2^ However, even when a randomised trial is feasible, ethical, sensible, well-planned and well-done, there are often important limitations, including when interpreting the findings—especially when the intervention or outcome is complex.^3^ For one, outcomes are predetermined and limited in number and might lack nuance.

Qualitative studies, particularly process evaluations, can offset some limitations of randomised trials.^3^,^6^ In qualitative studies, researchers can follow emerging lines of inquiry throughout data collection, collect detailed data about participants’ and investigators’ experiences of what happened and explore potential mechanisms of effects.^3^,^6^ This is especially important when there is little evidence about what outcomes are relevant or how to reliably measure them. A systematic review by MO *et al* has shown there is a lack of such evidence for interventions to improve critical thinking about health choices (submitted). The review was not registered. It was a partial update and pragmatic, ad hoc extension of an earlier review (CRD42016033103).^7^

By health choices, we mean decisions about what to do for the health of individuals or groups, that is, decisions about health interventions. When making health choices, people need critical thinking skills to avoid acting on unreliable claims and evidence about the effects of health interventions.^2 8^ People’s lack of ability to think critically about health choices^9 10^ can help explain widespread overuse of ineffective and harmful health interventions,^11^,^13^ as well as underuse of effective health interventions,^14^ including in low-income countries,^12 13^ where there are few resources to waste.

There are many definitions of critical thinking.^15^ A succinct definition consistent with how we use the term is ‘reasonable reflective thinking focused on deciding what to believe or do’.^16^ More specifically, in the Informed Health Choices (IHC) project, we have defined critical thinking about health choices as the ability to apply the essential concepts (principles) in the IHC key concepts framework.^2^ In terms of critical health literacy, applying the IHC key concepts is part of information appraisal.^17 18^ The last version of the framework includes 49 concepts (eg, that health interventions can have adverse effects), and four groups each of competencies (eg, the ability to question the basis for claims about the effects of health interventions) and dispositions (eg, being in the habit of questioning the basis for such claims).

In an earlier study, we designed an intervention to improve secondary school students’ ability to apply nine of the concepts in the framework and ‘transfer’^19^ those skills from the lessons to their daily lives: the IHC secondary school intervention.^20^ The intervention involved providing teachers with a 2–3-day training workshop and digital classroom resources, including lesson plans for 10 lessons to be delivered over the course of one semester. The intervention was effective in terms of the primary outcome measure, which was passing a multiple-choice test (≥9 of 18 questions answered correctly), although the effect was smaller and the certainty of the evidence lower after 1 year.^21^

Like health interventions, interventions to improve critical thinking about health choices can have adverse effects. An adverse effect (harm) of an intervention is an increase in adverse (undesirable) outcomes or decrease in beneficial (desirable) outcomes caused by the intervention. Researchers generally do not consider or assess potential adverse effects of interventions to improve critical thinking about health choices, as shown in the systematic review by MO (Matt Oxman) *et al.*

We assessed intended effects of the IHC secondary school intervention in randomised trials in Kenya, Rwanda and Uganda.^21^,^24^ After 1 year, we also assessed adverse outcomes in the intervention arms.^21^ In mixed-methods process evaluations in each country, we explored potential benefits of the intervention, both intended and unintended, and factors related to implementation.^25^,^27^ In this qualitative study, the objective was to explore participants’ experiences of potential adverse effects, across the three trial settings, and—based on findings about participants’ experiences—revise a framework of potential adverse effects of interventions to improve critical thinking about health choices, for use in future research.

## Methods

### Study design

This study was a combined process evaluation across the three trial sites, in Kenya, Rwanda and Uganda, based on data from the country-level process evaluations.^25^,^27^ This design was unique. While individual patient data meta-analyses are an established study design in quantitative research,^28^ we are unaware of any other qualitative study that involved synthesising data, not findings. Table 1 provides an overview of the development and evaluation of the IHC secondary school intervention, including the trials, country-level process evaluations and this study.

**Table 1:** Overview of the development and evaluation of the intervention

Period		Study	Methodology	Objectives
2019	2022	Development of intervention^20^	Mixed	Develop effective, desirable and feasible intervention
“	“	Development of initial framework of potential adverse effects^41^	“	Inform development and evaluation of intervention
2022	2023	Randomised trials and meta-analyses:	Quantitative	Assess effects of intervention
		Initial assessments (immediately following school term in which intervention was delivered)^22^,^24^		Assess intended effects
		1-year follow-up assessments^21^		Assess intended effects Assess adverse outcomes in intervention arms
“	2024	Country-level process evaluations^25^,^27^	Mixed	Explore potential benefits and factors related to implementation
“	2025	Cross-trial process evaluation (this study)	Qualitative	Explore experiences of potential adverse effects

Online supplemental file 1 is the protocol for this study, which we originally published in *Zenodo*.^29^ For an explanation of why we went away from calling the study a qualitative evidence synthesis, see the section on deviations from the protocol. Online supplemental file 2 is the Standards for Reporting Qualitative Research reporting checklist^30^ for this study. We used the checklist when editing the manuscript.

### Reflexivity

We applied both individual and team-reflexive strategies in the country-level process evaluations and in this study. The primary investigators in the country-level process evaluations (FC, MM and RS) and in this study (MO) were in special positions to influence the conduct and reporting of those studies, because they collected data and led the analysis, and therefore, applied reflexivity individually. In the country-level process evaluations, the primary investigators kept reflexive notes. In this study, the primary investigator kept reflexive notes and wrote a reflexive autobiography while planning, conducting and writing up the study. We had two team-reflexive discussions and agreed on a team-reflexive statement, reported as a supplement to the country-level process evaluations.^25^,^27^

### Study settings and participants

The trials were in secondary schools in five randomly sampled subcounties of Kisumu County in Kenya; districts representing all five provinces in Rwanda; and six districts in the central region of Uganda.^22^,^2527^ For the interviews for the process evaluations, each country team recruited students and teachers in the intervention arm, as well as other adult interest-holders,^31^ including parents of students in the intervention arm, school administrators and policy-makers.^25^,^27^ The teams purposively sampled schools and students to ensure variations in terms of schools and sociodemographic characteristics, including gender. In all countries, the interviewed students were adolescents aged ≥13 years. For more details about the study settings and participants in each country, see the reports of the country-level process evaluations^25^,^27^ and trials,^22^,^24^ as well as mixed-methods studies of the contexts for the intervention, conducted at the start of the project.^32^,^34^

### Data collection and extraction

The process evaluations involved three methods of qualitative data collection: (1) surveying teachers in the intervention arms of the trials using evaluation forms for the training and lessons, including qualitative (open-ended) items; (2) observing lessons and (3) interviewing participants in person, in groups (students and teachers) and individually (teachers and other adult participants), between the initial and 1-year follow-up assessments for the trials. The primary investigators in the country-level process evaluations and this study, as well as local research assistants, collected the data (administered evaluation forms, took observation notes and conducted interviews). The evaluation forms and original interview guides are included as supplementary files with the protocols for the country-level process evaluations.^35^,^37^ The authors of the protocols developed the forms and guides. Interviewers used separate, semistructured interview topic guides for different interest-holders. Over the course of the process evaluations, we adjusted the guides. We audio-recorded all interviews and transcribed the recordings for coding. We expected interviews to last 1–1.5 hours. When interviewing participants, we typically used the term ‘disadvantage’ as a plain-language alternative to ‘adverse effect’. For this study, the primary investigators in the country-level process evaluations extracted relevant, qualitative data from evaluation forms, observation notes and interview transcripts. MO combined those data in a spreadsheet for the analysis in this study.

### Modified framework analysis

We conducted a modified framework analysis as described by Ritchie and Spencer.^38^ In their description, framework analysis has five overlapping and iterative stages: ‘familiarisation’, ‘developing a thematic framework’, ‘indexing’, ‘charting’ and ‘mapping and interpretation’.^38^ They note that the nature of the final stage will depend on the original objectives, as well as what emerges from the data.^38^ In this study, the final stage involved what Ritchie and Spencer call ‘defining concepts’ or ‘clarifying definitions’^38^—specifically, defining potential adverse effects.

We did not abstract or synthesise data in charts or by participant because of the large number of participants. Therefore, we called the fourth stage ‘abstraction and synthesis’, rather than ‘charting’. We also changed the title of the fifth stage, calling it ‘revising the framework’, which is more specific than ‘mapping and interpretation’.

#### Development of the initial framework

We iteratively developed an initial framework based on two earlier frameworks,^39 40^ internal discussions and feedback from researchers and others with a variety of relevant expertise.^41^ The framework included categories of adverse outcomes (eg, equity harms); specific outcomes within each category (eg, benefit-based inequity); suboutcomes (eg, benefit-based inequity between students depending on a priori academic ability); categories of individuals or groups that might be directly or indirectly affected (eg, participants or their families) and corresponding beneficial outcomes (eg, benefit-based equity). We describe the development of the initial framework in detail in a separate report.^41^ Because the framework is ultimately intended to help measure potential effects, its terminology is largely quantitative.

#### Familiarisation with the data

This study involved two stages of familiarisation. First, as part of the country-level process evaluations, at least two investigators in each country familiarised themselves with all data collected for those studies, by reading and re-reading the transcripts and other data.^25^,^27^ Second, MO familiarised himself with data extracted for this study, by reading and re-reading the data (see ‘Data collection and extraction’) and relevant parts of transcripts.

#### Indexing

Like with familiarisation, this study involved two stages of indexing. First, two investigators in each country did an initial, rough coding of data collected for the country-level process evaluations. Second, MO fine-coded the data extracted for this study, assigning layers of themes to each extract. If MO was unsure about how to code an extract or whether to include it in the analysis, he consulted at least one of the other investigators.

Table 2 shows the initial coding scheme, which had two layers: initial themes, representing categories of potential adverse effects (eg, psychological harms) and initial subthemes, representing adverse outcomes within those categories (eg, work/schoolwork-related stress). The initial coding scheme was based on the initial framework.^41^

**Table 2:** Initial coding scheme based on the initial framework^41^

Initial theme: category of adverse effect	Description of initial theme: definition of category	Initial subtheme: adverse outcome	Description of initial subtheme: definition of adverse outcome
Decision-making harms	Behaviours and beliefs that might contribute to poor choices	Misunderstanding	Incorrect understanding of a concept or example explained in the intervention
		Misapplication	Incorrect or unnecessary application of a skill or knowledge learnt from the intervention
Psychological harms	Uncomfortable thoughts and feelings	Distrust	Feeling that a person or organisation cannot be relied on
		Pessimism	Inclination to believe that the application of skills or knowledge learnt from the intervention is impossible or useless
		Cognitive dissonance	Experience of inconsistent beliefs
		Stress	Mental or emotional strain from work or schoolwork
Equity harms	Inequities in the distribution or size of effects	Benefit-based inequity	Inequity due to the distribution or size of a beneficial effect of the intervention
		Harm-based inequity	Inequity due to the distribution or size of an adverse effect of the intervention
Group and social harms	Harmful interactions between individuals, groups, populations, and systems	Conflict	Unconstructive argument between two or more parties
Waste	Waste of time and resources	Wasted time	Use of time on the intervention that would be better spent on other activity
		Wasted resources	Use of resources on the intervention that would be better spent on other activity

Based on emerging findings, MO made changes to the initial coding scheme. He discussed changes with ADO. We added a third layer of themes to each extract and a fourth layer to some extracts. The additional layers represented suboutcomes. For example, within group and social harms, for extracts linked to conflicts, we differentiated conflicts between students and adults from conflicts between students and peers, and further between different populations of adults or peers (eg, parents or teachers). If what was said in an extract was too unclear to meaningfully be assigned a theme or did not appear to be about potential adverse effects of the intervention, MO excluded the extract from the analysis. He discussed exclusions with ADO. We also coded for ‘type’ of data: whether an extract described a participant’s experience, observation or opinion, or an investigator’s observation. And we coded for interpretation: whether the participant described a potential effect as adverse, or we interpreted it as such.

#### Abstraction and synthesis

We abstracted and synthesised the data, generating findings, in four steps: (1) creating a document with headings for each subtheme; (2) sorting the fine-coded data in the spreadsheet by the three levels of theme; (3) pasting data from the spreadsheet under the appropriate heading in the document and (4) abstracting and synthesising data within each subtheme in the document. The fourth step involved summarising similar participant experiences by rewording or paraphrasing the data, leaving some quotes from interview transcripts as verbatim examples. Finally, we compared the findings to the raw data, to ensure each data point was captured by the relevant finding, and that the data were captured accurately. This led to both adjusting the wording and structure of some findings, changing the coding of some extracts and excluding extracts for being too unclear to meaningfully code.

Based on the codes for different types of data (see ‘Indexing’ above), we noted which findings were largely based on participants’ explicit interpretations of a potential effect being adverse and which were largely based on our interpretation. This corresponds with distinguishing between first and second-order constructs, as defined by Atkins *et al*,^42^ based on theory developed by Schutz^43^; first-order constructs ‘reflect participants’ understandings’, while second-order constructs are ‘interpretations of participants’ understandings by study authors’.^42^

### Revising the framework

Based on the findings about each potential adverse effect, as well as reflection and discussion, we revised the initial framework. Specifically, we defined or redefined categories of potential adverse effects of interventions to improve critical thinking about health choices and defined potential adverse effects within those categories.

### Patient and public involvement

The design and conduct of this study was informed by earlier studies in which we interviewed and surveyed secondary school students, teachers and other interest-holders in Kenya, Rwanda and Uganda about the context for the intervention,^32^,^34^ the content and design of the intervention^20^ and the content of the initial framework used in the analysis in this study.^41^ For example, in those studies, participants expressed concern about the intervention potentially contributing to work or schoolwork-related stress—a potential harm that we then explored in this study. To inform the development and evaluation of the IHC secondary school intervention as a whole (table 1), we established national advisory networks in Kenya, Rwanda and Uganda, at the start of the project, including respective networks of students, teachers and policy-makers.^44^ We regularly sought input from the networks. However, neither students, teachers nor other members of the public were directly involved in the production of the protocol for this specific study, nor the conduct or reporting of the study. The primary investigators in the country-level process evaluations have organised in-person meetings to present results of the greater project to interest-holders in each country.

### Consent

In Kenya and Uganda, we obtained consent from school principals or head teachers at each school in loco parentis on behalf of their students. In all countries, we obtained informed assent from all students and informed consent from all other participants.

### Deviations from the protocol

We published the protocol for this study in *Zenodo*.^29^ In the protocol, we called the study a qualitative evidence synthesis, since the analysis would be of data from three corresponding, but separate, country-level process evaluations. However, it is more accurate to call it a cross-trial process evaluation since the study involved the primary analysis of primary data. Because of limited data and limits to the quality of the data, we did not explore differences across trial settings (see discussion). Because we no longer conceptualised the study as a qualitative evidence synthesis, we did not apply the Grading of Recommendations Assessment, Development and Evaluation–Confidence in Evidence from Reviews of Qualitative research approach.^45^

## Results

Table 3 is an overview of study participants across the country-level process evaluations. We only interviewed teachers individually in Rwanda and Uganda (not in groups), and only interviewed groups of parents in Kenya and Rwanda. We have structured the rest of the results in two subsections: the revised framework and findings about experiences of potential adverse effects. We present the changes to the framework first, since it includes definitions underlying findings in the second subsection.

**Table 3:** Overview of study participants across the country-level process evaluations

	Total	Kenya^25^	Rwanda^27^	Uganda^26^
Survey				
Workshop evaluation	121 teachers	39 teachers	42 teachers	40 teachers
Lesson evaluation (≥1 of 10 forms)	122 teachers	40 teachers	42 teachers	40 teachers
Observation (≥1 lesson)	96 classes	40 classes	16 classes	40 classes
Interviews				
Students	30 groups	10 groups	10 groups	10 groups
Teachers	2 groups 28 individually	2 groups 8 individually	0 groups 10 individually	0 groups 10 individually
Parents	8 groups 3 individually	2 groups 0 individually	5 groups 0 individually	1 group 3 individually
School administrators	19 individually	5 principals	10 head teachers or directors of studies	4 head teachers
Other interest-holders	20 individually	6 curriculum developers 3 policy-makers	2 policy-makers	9 policy-makers

### Revised framework

Table 4 shows the first part of the revised framework, which is the categories of potential adverse effects. Table 5 shows the second part, which is the adverse effects within those categories, with definitions. To avoid unnecessary wordiness, we use arrows to indicate the direction of effects (increase or decrease). The corresponding tables in the initial framework contained categories of adverse outcomes, as opposed to effects and outcomes within those categories. However, as exemplified in this study, adverse effects can also be decreases in beneficial outcomes (see discussion). Another realisation made during this study was that some outcomes in the initial framework are not necessarily adverse in and of themselves. In these cases, we revised the title or definition of the outcome. For example, we changed ‘Cognitive dissonance’ to ‘Adversely experienced cognitive dissonance’, and we define conflict as an interaction ‘ending in psychological or physical harm’.

**Table 4:** Part I of revised framework: categories of potential adverse effects of interventions to improve critical thinking about health choices

Category of adverse effect	Definition of category	
	Direction of effect	Definition of adverse or beneficial outcomes
Decision-making harms	↑	Behaviours or beliefs that can contribute to inappropriate choices
	↓	Behaviours or beliefs that can contribute to appropriate choices
Psychological harms	↑	Mental or emotional harm
	↓	Mental or emotional well-being
Equity harms	↑	Unfair distribution or size of benefits or harms across different groups
	↓	Fair distribution or size of benefits or harms across different groups
Group and social harms	↑	Interactions between individuals, groups, or systems contributing to adverse outcomes
	↓	Interactions between individuals, groups, or systems contributing to beneficial outcomes
Waste	↑	Inappropriate use of time or resources
	↓	Appropriate use of time or resources
Other harms	↑	Other adverse outcomes
	↓	Other beneficial outcomes

↑=Increase in outcome.

↓=Decrease in outcome.

**Table 5:** Part II of revised framework: potential adverse effects of interventions to improve critical thinking about health choices

Category of adverse effect	Adverse or beneficial outcome	Definition of adverse effect	
		Direction of effect	Definition of adverse or beneficial outcome
Decision-making harms	Adverse misunderstanding	↑	Incorrect understanding of what someone was intended to learn, which can contribute to inappropriate choice
Psychological harms	Anxiety related to transfer of learning	↑	Worry that transfer of learning will be difficult or harmful
	Adversely experienced cognitive dissonance	↑	Perceived inconsistency between beliefs that is experienced as adverse
	Work or schoolwork-related stress	↑	Mental or emotional strain from work or schoolwork
Equity harms	Inequity	↑	Unfair distribution or size of benefit or harm across different groups
	Equity	↓	Fair distribution or size of benefit or harm across different groups
Group and social harms	Conflict	↑	Disagreement ending in psychological or physical harm
Waste	Inappropriate use of time or resources	↑	Use of time or resources that is considered ineffective, inefficient or unimportant
	Appropriate use of time or resources	↓	Use of time or resources that is considered effective, efficient and important
Any category above or other	Other adverse outcome	↑	Adverse outcome not specified above
	Other beneficial outcome	↓	Beneficial outcome not specified above

↑=Increase in outcome.

↓=Decrease in outcome.

In the revised framework (table 5), unlike in the initial framework (table 2), we do not specify an increase in distrust of people or organisations, or an increase in misapplication of learning. We did not find any indication in the data, explicit or implicit, that the intervention potentially caused distrust that was somehow adverse. However, the revised framework does not rule out adverse effects involving trust or distrust, as they are implicitly captured as an increase in ‘other adverse outcome’ or decrease in ‘other beneficial outcome’ (table 5). We had defined misapplication as someone correctly understanding something they were intended to learn but applying it unnecessarily—‘overtransfer’^19^—or incorrectly, due to limitations of the intervention being unclear.^41^ However, if such a limitation is unclear, the person has misunderstood the extent of what they have learnt. Therefore, we conceptualised such events as a type of misunderstanding, rather than misapplication.

### Decision-making harms

Many participants—almost all students—demonstrated adverse misunderstandings of what they were intended to learn from the intervention. These findings are based on our interpretation of participants’ experiences, sometimes differing from the participant’s own explicit interpretations. However, a few participants explicitly suggested the intervention caused or might have caused adverse misunderstandings.

Many students described unreliable claims as facts or advice that they had learnt from the intervention, and many of those students said they had acted on such claims. Some of those claims were examples of unreliable claims in the resources, particularly the claim that toothpaste cures pimples. Others were not, such as the claim that flour or egg yolk heals burns.

She [the teacher] told us that some people use Colgate on their face when they have pimples. So, I went home and tried it, but it did not work. —Student

Let’s say I’m cooking then I burn myself. Before I learned this lesson, I used to put my hand in cold water. After I put my hand in cold water, the wound still formed, and it was painful. But after learning this lesson, I learnt that I can either put flour on it or I can use an egg yolk, and it will heal. —Student

Similarly, some students demonstrated complete misunderstandings of concepts when describing what they had learnt from the intervention—for example, the concept that claims about the effects of health interventions are unreliable if only based on how widespread the claim is.

I learnt about critical thinking […] For example, using charcoal to heal [a stomach condition], you need to first make proper research to know whether to use it. If there are many who tell you that it works, then you can go ahead. If there are few, you pick another option. — Student

In terms of competencies, some students incorrectly suggested they had learnt to search for information about the effects of health interventions—the reliability of which they would be unable to assess with just the skills they were intended to learn.

[…] I learnt about critical thinking, and Madam [the teacher] taught us you must think critically before doing something. When they say that, ‘Today, we are going to be vaccinated,’ let me say [against] hepatitis, I must first research it and say, ‘That vaccine, how many people died?’ or ‘How many people are still alive?’ before I take it. —Student

Some students suggested they had learnt to measure the effects of health interventions themselves, either by testing the intervention on themselves or conducting a trial—as opposed to assessing or using evidence from studies conducted by professional researchers. Sometimes, students said they had acted on such misunderstandings.

[…] the most interesting thing I learn was to carry out experiments. In case you want to treat someone when you are seeing those tablets, you can first get those tablets and do the experiment. —Student

Some misunderstandings were of the extent of a concept or competency that students were intended to learn. For example, one teacher expressed concern that students would understand that study samples must be large enough to avoid chance results but mistakenly think that they have learnt how to assess when a sample is large enough.

Finally, both a policy-maker and a director of studies suggested the intervention might cause overtransfer of critical thinking in students, as opposed to prioritising when to think critically. One student described experiencing overtransfer.

Let’s say there is an example of a spoon here on the table, and then I am like, should I take it, or shouldn’t I take it? So, it was wasting a lot of my time, overthinking everything that I do. —Student

### Psychological harms

#### Anxiety related to transfer of learning

In interviews, some students expressed worry that transferring what they had learnt from the intervention would be difficult or harmful when making health choices for themselves, in daily life. They based such concerns on their limited ability to make informed choices, limited resources or time, or peer pressure. Some of the same students and others expressed similar worry about helping other people, based on other peoples’ limited knowledge or ability to learn, limited access to resources or entrenched beliefs.

Studying these lessons is not difficult but applying them in real life is not easy. […] Sometimes it is easier to do what someone told you because you don’t have time to think critically about it or to consult people who are experts in that […] let’s say, for example that a person arrived at a health center and one of his or her friends tells them to do something, and I who studied this tell them to do another thing based on what I learned. They would not opt for my solution […] it necessitates to learn special lessons. (translated) —Student

A small number of students expressed worry that applying what they had learnt could prevent them from doing something helpful for their health or cause them to prevent someone else from doing something helpful, because the only information they have is unreliable.

After I learned that there are reliable and unreliable claims, I had this confusion, thinking that I will sometimes refuse to take a medicine that may be helpful, thinking that the medicine is not reliable due to weak bases. And this has created some confusion in me because I may sometimes make myself suffer by not taking the adequate medicine for my illness. (translated) —Student

#### Adversely experienced cognitive dissonance

A few students and a teacher said they had adverse experiences of inconsistent beliefs related to the intervention. Some of the inconsistent beliefs were about key concepts explained in the intervention, specifically those introduced in the lessons about unreliable claims, including claims based only on how long or how many people have used an intervention.^20^

[…] my grandmother influenced me a lot to believe that when something has been used for a long time, it is reliable. But when I came to this lesson, I learnt that it is not reliable, but although my grandmother and everybody uses that medicine, and it cures them. So, I’m a bit confused also. —Student

Other inconsistent beliefs were about claims themselves. Some of those claims were examples of unreliable claims used in the resources—particularly the claim that applying toothpaste to a pimple cures the pimple. Others were not used as examples in the resources—for example, that taking ginger relieves a cough. In the latter cases, the source or basis of the claim was often unclear.

The main disadvantage [of the intervention] is that it can be challenging to make a comparison to what you already knew, and now they are telling you that it is not true. If someone comes and tells you that ginger cannot treat cough while you know that it treats it, it can be difficult to understand it. (translated) —Student

#### Work or schoolwork-related stress

In interviews and via a lesson evaluation form, some students and teachers said the intervention had caused them stress. Generally, the stress was related to time pressure or to the complexity of the content.

When we joined this Form 1, we were introduced to 11 subjects. Then, there was a time that we were told by our teacher that we are going to have an additional subject, which was IHC [Informed Health Choices], so at that time we had not yet adapted to the 11 subjects and yet they had another subject that we were to do […] that was stressful and also tiresome. —Student

Teachers said the content was difficult to teach because some concepts were difficult to understand or explain—particularly the concepts about randomisation and random error—or because the content prompted difficult questions from students about applying what they had learnt and caring for their health.

[…] I became a school nurse. So, I was receiving a lot of questions from the students. Sometimes, they could go to the hospital, they are given drugs, and they rush to me; ‘Is it OK with us? You told us that we should not be taking painkillers all the time, but we have been given a lot of them. Is it OK?’ So, it was a bit challenging to convince them just to take what the doctors have prescribed. —Teacher

#### Equity harms

A small number of students and teachers suggested the intervention might cause inequities (ie, decrease equity) or exacerbate inequities (ie, increase inequity). Two students suggested that the knowledge or skills learnt from the intervention might not benefit people with limited access to resources or might benefit them less than people with better access, in terms of opportunities to use the knowledge or skills.

What I think is a disadvantage of learning these lessons is that you sometimes couldn’t find where to get the reliable health information. For example, people living in villages may not have necessary tools to access the information easily. People living in cities may have better access to reliable health information than those living in villages. (translated) —Student

Two teachers suggested the intervention might cause inequities depending on the students’ a priori abilities, either ‘scaring’ students who already struggle with mathematics (a harm-based inequity), or being of greater benefit to students with stronger academic abilities at the start of the intervention.

You find students with a higher ability together in the same class as those with a lower ability. […] Most of the time we randomly create the classes. So, when you are teaching this, there is most likely a case where you will have those students who will understand more than the others. So, I think because of the learning differences between students, inequity is highly likely to be a disadvantage that will be experienced. —Teacher

### Group and social harms

In interviews, there were many participants who attributed conflicts to the intervention or said the intervention might cause conflicts. This included students and teachers who received the intervention, parents of students who received the intervention, administrators at intervention schools and policy-makers. Some data provided by teachers via lesson evaluation forms, as well as some observation notes made by investigators, also suggested the intervention caused conflicts. In most cases, the harm was psychological.

[…] students from other classes wouldn’t take us seriously when you came and told them that what they are thinking is wrong. […] and that discouraged us. (translated) —Student

In a group interview, two students said disagreements with their parents ended in corporal punishment. In other interviews, participants said disagreements had ended in threats of corporal punishment.

It affected me negatively because my mom used to give me the traditional herbs and [after the IHC secondary school intervention] I didn’t want to take them, so my mum and I fought sometimes. Sometimes she would beat me. —Student

All but one conflict described by participants involved students who received the intervention. The exception was a conflict between a teacher and the teacher’s mother about a health claim made by the mother based on anecdotal evidence.

Participants described conflicts between students and adults or peers, or both. The adults were often parents, but also included other family members, teachers and others, such as herbalists. The peers included classmates (students who also received the intervention), schoolmates (students at the same school who did not receive the intervention), friends and siblings.

Many of the disagreements were around ‘traditional’ or ‘herbal’ care.

She didn’t allow me to give my child an herbal medicine and took it from the fire where I was warming it and threw it away. That made me sad, and I asked her, ‘Are you going to have rules in my house more than me? I gave birth to you, and you are going to direct me?!’ It made me angry and if I was the same person I used to be, I could have beaten her up, but it required me to take time and think about it, because I could not understand how those medicines that I used for so long to treat all my children, and I warm them, why she could take them and throw them away. You understand that it was going to bring a fight, but I managed it by keeping my distance from her. But if I had stayed and we continued to see each other in the eyes, it could be fights (translated) —Parent

One of our friends had passed away, so we went for the burial, and as you know people take advantage of such environments to sell their stuff. Then came a vendor who had drugs that stop one from vomiting […] I told my brother to first think through before buying a drug [that] stops vomiting […] He told me that I was de-marketing the guy, so he promised to slap me, and I went on and told him that he can go on and do it, but I stand by my word that the drug doesn’t work, because the basis of the claim is weak. The man too joined the quarrel saying that I was demarketing him. At the end of it all, my brother didn’t buy the drug, but the man wanted to beat me because I was talking the facts. —Student

### Waste

At some schools, the intervention took time from physical education, and a small number of teachers suggested students would have preferred the latter, that is, that students experienced the intervention as relatively unimportant.

I’m very sure that even there are some students who will still feel that why are we not given this time to go out and play when it is time for P.E. —Teacher

## Discussion

In this study, we found that participants in trials of the IHC secondary school intervention in Kenya, Rwanda and Uganda experienced or anticipated potential effects of the intervention that they described as adverse, or that we interpreted as such. These include decision-making harms, psychological harms, equity harms, group and social harms, and waste. Informed by the findings, we revised our initial framework of potential adverse effects of interventions to improve critical thinking about health choices.

We analysed data from a variety of participants—including students, teachers, parents and policy-makers—across three trial settings and countries, collected using a variety of methods—including survey (open-ended items), observation and individual and group interviews. To collect and analyse the data, we used an iteratively developed framework,^41^ which pointed us in fruitful directions of inquiry. We applied both individual and team-reflexive strategies.

Limitations of this study include that it only provides a snapshot of participants’ experiences and views. Adverse effects might only be short-term or first occur long-term. For example, students’ anxiety related to applying what they have learnt might be temporary and wear off with reflection, practice and maturation. On the other hand, with time, more students might recognise the limitations of what they have learnt and become anxious about using it. Also, we have not explored the importance that interest-holders place on different effects. Some of the potential adverse effects—such as an increase in adversely experienced cognitive dissonance—might be considered unavoidable, short-term effects of learning that are outweighed by long-term benefits.

The initial framework only included adverse effects that would be increases in adverse outcomes, but adverse effects can also be decreases in beneficial outcomes. A potential decrease in beneficial outcomes caused by the IHC secondary school intervention is, for example, a decrease in placebo effects of otherwise ineffective interventions with negligible harms (eg, drinking hot tea with lemon to prevent the common cold). We did not explore experiences of this potential effect. However, it is implicitly included in the revised framework as a decrease in ‘Other beneficial outcome(s)’ (table 5).

We did not explore differences across different contexts or populations either. It is possible that there are important differences. For example, it is possible that participants with a lower level of English across the three countries, or participants in Rwanda, where English more recently became the main language of education, had experiences that were different from those of other participants, given that teaching resources were in English except for translations of some key terms.

The earlier-referenced systematic review by MO *et al* (in production) was of quantitative studies of interventions to improve critical thinking about health choices (see introduction). However, it also included process evaluations or other qualitative studies associated with the included quantitative studies, in which researchers explored potential adverse effects of the intervention.^4046^,^48^ The findings from those qualitative studies are too limited in number, and the reported data too limited in richness, for a meaningful synthesis with findings from this study. However, there are some apparent similarities. For example, along the lines of our findings about conflict, and those about anxiety about transfer of learning, Berger *et al* found some self-help group members expressed ‘discouragement’ about transferring skills they had learnt from an evidence-based medicine training course because they had experienced ‘negative reactions from professionals if they raised critical questions concerning therapeutic issues’.^46^

Our study highlights the need for programme or intervention designers to consider potential adverse effects of interventions to improve critical thinking about health, as well as how to mitigate harms. For example, in the piloting phase, they can interview participants about potential harms and mitigation strategies. The study also highlights the need for educators and policy-makers to consider trade-offs between potential harms and potential benefits of the interventions.

New studies can build on the findings of this study, as well as address its limitations. For example, they can explore interest-holders’ perspectives of the importance of different adverse effects or explore experiences of potential adverse effects within other contexts (eg, high-income countries) or across different contexts (eg, low-income vs high-income contexts) or populations (eg, across interest-holders). Furthermore, the findings of the study can be used as a starting point for developing quantitative measures of adverse outcomes and developing and evaluating strategies for mitigating adverse effects.

## Conclusions

By exploring participant experiences, this study provided valuable insight into potential adverse effects of interventions to improve critical thinking about health choices, including decision-making harms, psychological harms, equity harms, group and social harms, and waste. The study complements the quantitative assessments of the IHC secondary school intervention,^21^,^24^ as well as the country-level process evaluations.^25^,^27^ Preliminary findings from this study informed quantitative assessments of adverse outcomes in the intervention arms of the trials after 1 year, as reported in a prospective meta-analysis.^21^ The revised framework might be useful in the design or evaluation of other interventions to improve critical thinking about health choices, as well as other educational interventions. Future research can build on this study, as well as address its limitations, for example, by exploring views on the importance of different adverse effects or exploring differences in experiences of potential adverse effects within or across different populations and contexts. The results of such research can be used to improve the framework.

## Supplementary material

10.1136/bmjopen-2025-104236 online supplemental file 1

10.1136/bmjopen-2025-104236 online supplemental file 2

## Data Availability

Data are available in a public, open access repository.

## References

[1] Oxman AD, Chalmers I, Dahlgren A (2023). Key concepts for informed health choices. 2.1: comparisons of treatments should be fair. J R Soc Med.

[2] Oxman AD, Chalmers I, Dahlgren A (2022). Key concepts for informed health choices: Where’s the evidence? [version 1; peer review: 1 approved with reservations]. F1000Res.

[3] Lewin S, Glenton C, Oxman AD (2009). Use of qualitative methods alongside randomised controlled trials of complex healthcare interventions: methodological study. BMJ.

[4] Doyle L, Brady A-M, Byrne G (2016). An overview of mixed methods research – revisited. J Res Nurs.

[5] Weiss C, Mosteller F, Boruch R (2002). Evidence matters.

[6] Bonell C, Jamal F, Melendez-Torres GJ (2015). ‘Dark logic’: theorising the harmful consequences of public health interventions. J Epidemiol Community Health.

[7] Cusack L, Del Mar CB, Chalmers I (2018). Educational interventions to improve people’s understanding of key concepts in assessing the effects of health interventions: a systematic review. Syst Rev.

[8] Chalmers I, Oxman AD, Austvoll-Dahlgren A (2018). Key Concepts for Informed Health Choices: a framework for helping people learn how to assess treatment claims and make informed choices. *BMJ Evid Based Med*.

[9] Boutron I, Haneef R, Yavchitz A (2019). Three randomized controlled trials evaluating the impact of “spin” in health news stories reporting studies of pharmacologic treatments on patients’/caregivers’ interpretation of treatment benefit. BMC Med.

[10] Dahlgren A, Furuseth-Olsen K, Rose CJ (2020). The Norwegian public’s ability to assess treatment claims: results of a cross-sectional study of critical health literacy [version 2; peer review: 1 approved, 1 approved with reservations]. F1000Res.

[11] Brownlee S, Chalkidou K, Doust J (2017). Evidence for overuse of medical services around the world. The Lancet.

[12] Albarqouni L, Palagama S, Chai J (2023). Overuse of medications in low- and middle-income countries: a scoping review. *Bull World Health Organ*.

[13] Albarqouni L, Arab-Zozani M, Abukmail E (2022). Overdiagnosis and overuse of diagnostic and screening tests in low-income and middle-income countries: a scoping review. BMJ Glob Health.

[14] Glasziou P, Straus S, Brownlee S (2017). Evidence for underuse of effective medical services around the world. Lancet.

[15] Geng F (2014). An Content Analysis of the Definition of Critical Thinking. ASS.

[16] Ennis RH (1989). Critical Thinking and Subject Specificity: Clarification and Needed Research. Educ Res.

[17] Oxman AD, García LM (2020). Comparison of the Informed Health Choices Key Concepts Framework to other frameworks relevant to teaching and learning how to think critically about health claims and choices: a systematic review. F1000Res.

[18] Chinn D (2011). Critical health literacy: a review and critical analysis. Soc Sci Med.

[19] Barnett SM, Ceci SJ (2002). When and where do we apply what we learn? A taxonomy for far transfer. Psychol Bull.

[20] Rosenbaum S, Moberg J, Chesire F (2024). Teaching critical thinking about health information and choices in secondary schools: human-centred design of digital resources [version 3; peer review: 1 approved, 2 approved with reservations]. F1000Res.

[21] Chesire F, Mugisha M, Ssenyonga R (2024). Effects of the informed health choices secondary school intervention after 1 year: a prospective meta-analysis using individual participant data. Trials.

[22] Chesire F, Kaseje M, Ochieng M (2023). Effects of the informed health choices secondary school intervention on the ability of students in Kenya to think critically about health choices: A cluster-randomized trial. J Evid Based Med.

[23] Ssenyonga R, Oxman AD, Nakyejwe E (2023). Use of the informed health choices educational intervention to improve secondary students’ ability to think critically about health interventions in Uganda: A cluster-randomized trial. J Evid Based Med.

[24] Mugisha M, Nyirazinyoye L, Simbi CMC (2023). Effects of the Informed Health Choices secondary school intervention on the ability of students in Rwanda to think critically about health choices: A cluster‐randomized trial. J Evidence Based Medicine.

[25] Chesire F, Oxman AD, Kaseje M (2024). Process Evaluation of Teaching Critical Thinking About Health Using the Informed Health Choices Intervention in Kenya: A Mixed Methods Study. Glob Health Sci Pract.

[26] Ssenyonga R, Lewin S, Nakyejwe E (2024). Process Evaluation of Teaching Critical Thinking About Health Using the Informed Health Choices Intervention in Uganda: A Mixed Methods Study. Glob Health Sci Pract.

[27] Mugisha M, Oxman AD, Nyirazinyoye L (2024). Process Evaluation of Teaching Critical Thinking About Health Using the Informed Health Choices Intervention in Rwanda: A Mixed Methods Study. Glob Health Sci Pract.

[28] Stewart LA, Clarke MJ (1995). Practical methodology of meta-analyses (overviews) using updated individual patient data. Cochrane Working Group. Stat Med.

[29] Oxman M, Oxman AD, Fretheim A (2023). Participants’ and investigators’ experiences and views of potential adverse effects of an educational intervention: Protocol for a qualitative evidence synthesis.

[30] O’Brien BC, Harris IB, Beckman TJ (2014). Standards for reporting qualitative research: a synthesis of recommendations. Acad Med.

[31] Akl EA, Khabsa J, Petkovic J (2024). “Interest-holders”: A new term to replace “stakeholders” in the context of health research and policy. *Cochrane Evid Synth Methods*.

[32] Ssenyonga R, Sewankambo NK, Mugagga SK (2022). Learning to think critically about health using digital technology in Ugandan lower secondary schools: A contextual analysis. PLoS One.

[33] Chesire F, Ochieng M, Mugisha M (2022). Contextualizing critical thinking about health using digital technology in secondary schools in Kenya: a qualitative analysis. Pilot Feasibility Stud.

[34] Mugisha M, Uwitonze AM, Chesire F (2021). Teaching critical thinking about health using digital technology in lower secondary schools in Rwanda: A qualitative context analysis. PLoS One.

[35] Chesire F, Kaseje M, Ochieng M (2022). Effect of the Informed Health Choices digital secondary school resources on the ability of lower secondary students in Kenya to critically appraise health claims: protocol for a process evaluation.

[36] Mugisha M, Nyirazinyoye L, Oxman A (2022). Use of the Informed Health Choices digital resources for teaching lower secondary school students in Rwanda to think critically about health: protocol for a process evaluation.

[37] Ssenyonga R, Lewin S, Nakyejwe E (2022). Informed heath choices intervention to teach secondary school adolescents in Uganda to assess claims about treatment effects: A process evaluation protocol.

[38] Ritchie J, Spencer L (1994). Qualitative data analysis for applied policy research. Analyzing Qualitative Data.

[39] Lorenc T, Oliver K (2014). Adverse effects of public health interventions: a conceptual framework. J Epidemiol Community Health.

[40] Nsangi A, Semakula D, Glenton C (2019). Informed health choices intervention to teach primary school children in low-income countries to assess claims about treatment effects: process evaluation. BMJ Open.

[41] Oxman M, Chesire FC, Mugisha M (2024). Development of a framework of potential adverse effects of interventions to improve critical thinking about health choices: A mixed methods study [version 1; peer review: 1 approved]. F1000Res.

[42] Atkins S, Lewin S, Smith H (2008). Conducting a meta-ethnography of qualitative literature: Lessons learnt. BMC Med Res Methodol.

[43] Schutz A (1974). Collected papers I. the problem of social reality.

[44] Nsangi A, Oxman AD, Oxman M (2020). Protocol for assessing stakeholder engagement in the development and evaluation of the Informed Health Choices resources teaching secondary school students to think critically about health claims and choices. PLoS One.

[45] Lewin S, Booth A, Glenton C (2018). Applying GRADE-CERQual to qualitative evidence synthesis findings: introduction to the series. Implement Sci.

[46] Berger B, Steckelberg A, Meyer G (2010). Training of patient and consumer representatives in the basic competencies of evidence-based medicine: a feasibility study. BMC Med Educ.

[47] Semakula D, Nsangi A, Oxman A (2019). Informed Health Choices media intervention for improving people’s ability to critically appraise the trustworthiness of claims about treatment effects: a mixed-methods process evaluation of a randomised trial in Uganda. BMJ Open.

[48] Steckelberg A, Fuhlendorf A, Jonitz C (2008). ebm@school – a curriculum of critical health literacy for secondary school students: results of a qualitative study to explore possible adverse effects.

[49] Oxman M (2025). Underlying data for "Participants’ experiences of potential adverse effects of an intervention to improve critical thinking about health choices: A cross-trial process evaluation. Zenodo.

