# Supplementary material for: Participants’ experiences of potential adverse effects of an intervention to improve critical thinking about health choices: a qualitative cross-trial process evaluation in Kenya, Rwanda and Uganda
Source: BMJ Open. 2025 Oct 21;15(10):e104236. doi: 10.1136/bmjopen-2025-104236 (PMC12548629; doi:10.1136/bmjopen-2025-104236)
Supplement: online supplemental file 1 [file bmjopen-15-10-s001.pdf]

Informed Health Choices

Participants’ and investigators’ experiences and views of potential adverse effects of an educational intervention: Protocol for a qualitative evidence synthesis

Oxman, M et al.

Working paper, February 2023

www.informedhealthchoices.org

Colophon

Title Participants' and investigators' experiences and views of potential adverse effects of an educational intervention: Protocol for a qualitative evidence synthesis

Authors Oxman, Matt
Oxman, Andrew D.
Fretheim, Atle
Lewin, Simon

Corresponding author(s) Oxman, Matt matt@mattoxman.com

Keywords adverse effects; harms; side effects; unintended effects; unanticipated effects; unexpected effects; education; students; pupils; learners; secondary school students; high school students; young people; youth; adolescents; school; secondary school; high school; educational interventions; public health interventions; teaching resources; learning resources; educational resources; secondary school resources; high school resources; critical thinking; critical appraisal; health literacy; critical health literacy; evidence-based healthcare; evidence-based medicine; health; public health; low-income countries; LICs; low and middle-income countries; LMICs; developing countries; qualitative evidence synthesis; QES; framework analysis; framework synthesis; "best fit" framework synthesis

Citation. Oxman *et al.* 2023. "Participants' and investigators' experiences and views of potential adverse effects of an educational intervention: Protocol for a qualitative evidence synthesis." *Zenodo*. DOI: 10.5281/zenodo.7681365.

Date February 2023

Abstract

Introduction

To help students think critically about health information and decisions, we developed the Informed Health Choices (IHC) secondary school intervention. We are evaluating the intervention in cluster-randomised trials, and linked process evaluations, in Kenya, Rwanda, and Uganda. The study planned herein is a qualitative evidence synthesis (QES), using data about experiences and views of adverse effects from all three process evaluations. The QES overlaps with the process evaluations. The QES will allow us to comprehensively explore, report, and discuss experiences and views of potential adverse effects, and potential mechanisms. The findings are intended to help inform decisions about whether or how to redevelop, re-evaluate, or implement the intervention. The methods and findings might also be helpful to developing, evaluating, or implementing other educational interventions—especially interventions intended to improve critical thinking, within health or other fields.

Objective

Explore participants' and investigators' experiences and views of potential adverse effects of the IHC secondary school intervention, and potential mechanisms of those effects.

Methods

An independent researcher will assess methodological limitations of the included studies, based on a list of domains used by the Cochrane Effective Practice and Organisation of Care (EPOC) group. We will merge and modify framework analysis as outlined by Ritchie and Spencer, framework synthesis as described by Barnett-Page *et al.*, and "best fit" framework synthesis as outlined by Carrol *et al.* All three are pragmatic approaches with a deductive analysis using an *a priori* framework, followed by an inductive thematic analysis. We will note possible differences in how participants generally conceptualise adverse effects of educational interventions, compared to the study team, and possible

differences in adverse effects across trial settings, for the purposes of future research. To assess confidence in the synthesis findings, we will apply the Grading of Recommendations Assessment, Development and Evaluation Confidence in Evidence from Reviews of Qualitative research (GRADE-CERQual) approach, using the interactive Summary of Qualitative Findings (iSoQ) tool. We will produce a Qualitative Evidence Profile, and Summary of Qualitative Findings Tables.

Discussion

The QES and the process evaluations overlapping and being part of the same project has methodological implications that amount to both strengths and limitations. Like in prospective meta-analyses, when planning the process evaluations, we harmonised the objectives, facilitating synthesis, while investigators in each setting still had autonomy to explore phenomena specifically for their study. Like individual patient data (IDP) meta-analyses, this study will be based on data from the process evaluations, facilitating more reliable analysis and synthesis than if it was only based on reported findings. As far as we are aware, this QES will be the first of its kind methodologically, and first empirical study of its size and rigour focusing on potential adverse effects of an educational intervention. The QES and process evaluations overlapping and being part of the same project also introduces risk of bias. Another challenge is that we are including the study team's experiences and views. In general, to address these challenges, we will be transparent, and apply reflexivity throughout.

Introduction

Adverse effects of educational interventions

People sometimes use the terms "effect" and "outcome" interchangeably, and each term can have different meanings in different contexts. Herein—based on the definition in the Glossary of Evaluation Terms for Informed Treatment choices (1,2)—an "effect" of an intervention is an increase or decrease in outcomes attributed to the intervention, while an "outcome" is an event, and the unit of measurement in quantitative studies. As follows, an "adverse effect" of an intervention is an increase in adverse outcomes, or decrease in beneficial outcomes, attributed to the intervention.

Herein, an "adverse effect" is synonymous with "undesirable effect", or "harm", although some people might understand "harm" as adverse effects on health outcomes only. "Adverse effect" is not synonymous with "unintended effect" or "unexpected effect", since a beneficial effect might also be unintended or unexpected. Importantly, "adverse" is relative: the same outcome or effect might be adverse to one individual or group, but not another.

Researchers assess the effects of interventions in randomised trials since randomisation is the only method of controlling for unknown confounding (3). In qualitative studies, researchers explore phenomena, often people's experiences and views (4). However, researchers can use qualitative studies—particularly process evaluations—to explore different stakeholders' experiences and views of *potential* effects, and potential mechanisms of effects (5–7).

Researchers often overlook adverse effects of educational interventions (8,9). When they do assess effects of educational interventions, they typically focus on intended effects (8,9).

Assessments of intended effects might reveal a lack of intended effect, or what in pharmacology are called "paradoxical" effects (10,11), herein a decrease in beneficial outcomes that the intervention was intended to increase, or an increase in adverse outcomes that the intervention was intended to decrease. For example, a randomised trial of the widespread school improvement programme Achievement for All, in the United Kingdom, showed a lack of

intended effect on certain attitudes, such as children's self-esteem, and a paradoxical effect on academic outcomes, such as progress in reading (12).

However, besides lacking intended effects or having paradoxical effects, educational interventions might have other adverse effects: "side effects" (8,9). For example, Zhao hypotheses that the widespread and costly American programme Reading First caused children to lose interest in reading due to the programme's emphasis on phonics (8,13).

In terms of this protocol, previous evaluations of educational interventions intended to improve laypeople's ability to think critically about health information and decisions are particularly relevant. In their systematic review focusing on such interventions, Cusack et al. (14) included 24 quantitative evaluations reported in 22 papers (15–36), with two of the papers reporting evaluations of the same intervention in two different populations (24,36). In updating the review, Verdugo-Paiva *et al*. (37) identified two follow-up evaluations (38,39), but no evaluations of additional interventions.

As part of preparing this protocol, we screened the papers included in the updated review and found one reported a quantitative evaluation of a potential adverse effect that would not be paradoxical (32). Another three (33,38,39) referred to linked qualitative evaluations that included views and experiences of potential adverse effects that would not necessarily be paradoxical (5,6,40). For example, in all three, investigators explored whether participants experienced conflicts with family after applying what they had learned, questioning family members' claims, beliefs, or choices.

It is ironic that evaluations of educational interventions intended to help people think critically about the benefits and harms of healthcare so often exclude any evaluation of potential adverse effects. In general, given standards for evaluating potential adverse effects in medical research (41,42), and historically the Hippocratic oath, one could logically expect evaluations of educational interventions within healthcare to be more likely to include potential adverse effects compared to evaluations of educational interventions within other fields. On the other hand, like with educational interventions, researchers have often overlooked the potential adverse effects of public health interventions (11,43).

The Informed Health Choices secondary school intervention

There is practically endless information about how best to care for our health, and a lot of it is unreliable (44). Many people are unable to assess the reliability of such information (45,46). Logically, this inability contributes to worldwide overuse of harmful or wasteful medical services (47–49), and underuse of

helpful services (50). The problem is most pressing in poor populations since the fewer resources you have, the fewer you can afford to waste.

Helping children and young people learn how to think critically about health information and decisions makes sense for several reasons (51,52). These include that they generally have more time to learn and build on what they learn, compared to adults, as well as less to unlearn.

To help students learn how to think critically about health information and decisions, we—a project team, including the authors of this protocol, and other researchers—developed the Informed Health Choices (IHC) secondary school intervention. We are evaluating the intervention in parallel cluster-randomised trials (53–55), and linked process evaluations, in Kenya, Rwanda, and Uganda (56–58). The trials include an assessment at the end of the school term in which participants received the intervention, and a one-year-follow-up assessment (53–55).

The secondary school intervention has two main components: a training workshop for teachers, and digital teaching resources. Prepared by the training and supported by the resources, teachers are intended to deliver 10 lessons to students within one school term. For a detailed description of the intervention, see any of the trial protocols (53–55).

In the trials of the secondary school intervention, we have found it challenging to measure adverse outcomes for a variety of reasons. These include limited time and resources for developing and evaluating (validating) outcome measures. Therefore, in the initial assessment of the trials, we only evaluated intended effects, like what we earlier described as typical for education research.

This study

The study planned herein is a qualitative evidence synthesis (QES) (59), using anonymised (de-identified) data from all three process evaluations (56–58). The aim is to explore participants' and investigators' experiences and views of potential adverse effects of the IHC secondary school intervention.

The QES overlaps with the process evaluations, i.e., the primary studies included in the QES (

Table 1). For the analysis and synthesis, we will use an *a priori* framework of potential adverse effects, which we developed in a separate study (60). Besides exploring experiences and views of potential adverse effects, the process evaluations include other objectives, and we are planning a separate QES of other data from the process evaluations.

Table 1. Studies included in evaluation of potential adverse effects of the IHC secondary school intervention, and related studies.

	Evaluation of potential adverse effects	Related studies
2019-2022	Development of *a priori* framework (60)	Development of intervention (60)
2022-2023	Process evaluations (56–58)[1,2]	Cluster-randomised trials (53–55):
	Qualitative evidence synthesis (this study)[1]	• Delivery of intervention
		• Initial assessment
		• One-year-follow-up assessment

[1]The process evaluations and this study overlap.
[2]Besides exploring experiences and views of potential adverse effects, the process evaluations include other objectives.

Rationale for this study

The QES will allow us to comprehensively explore, report, and discuss experiences and views of potential adverse effects of the IHC secondary school intervention, and potential mechanisms of adverse effects. Given the challenges to reliably measuring adverse outcomes in the trials, the QES is particularly important. The QES will inform the development of outcome measures for use in the one-year-follow-up assessment of the trials.

Beyond the trials, the QES findings are intended to help inform decisions about whether or how to redevelop, re-evaluate, or implement the IHC secondary school intervention. Beyond the project, the methods and findings might be helpful to researchers and others developing, evaluating, or implementing other educational interventions—especially interventions intended to improve critical thinking, within health or other fields.

Objective

Explore participants’ and investigators’ experiences and views of potential adverse effects of the IHC secondary school intervention, and potential mechanisms of those effects.

Ethics

The stages of this study that do not overlap with the process evaluations will not involve collecting new data. Rather, the only data used in this study will be anonymised data from the process evaluations—i.e., data from which investigators in the process evaluations have already removed any information that could be used to identify participants. For information about data collection, management, and privacy in the process evaluations, see the protocols for those studies (56–58).

The Norwegian Institute of Public Health (NIPH) is the lead partner in the development and evaluation of the intervention. As required by the institute—to comply with the European General Data Protection Regulation—we have completed a data protection impact assessment for the entire evaluation stage, including the process evaluations. The Data Protection and Chief Information Security Officers at the institute reviewed the assessments, and the relevant executive officer approved them.

As required by the funder, the Research Council of Norway, we have also created a data management plan for the entire project, which we are updating continuously, and will submit to the council at the end of the project.

Since the project will not generate new knowledge about health or disease, it falls outside the remit of the Regional Committee for Medical Research Ethics (61), in Norway, which the committee has confirmed (their reference: 30713).

In Kenya, we obtained ethics approval from Masinde Muliro University of Science and Technology Institutional Ethics Review Committee and the Kenya National Commission of Science and Technology Institute, as well as approval from the Ministry of Education and the Teachers Service Commission, nationally and at the county-level. In Rwanda, we obtained ethics approval from the Rwandan National Ethics Committee. In Uganda, we obtained ethics approval from the Faculty of Medicine Research and Ethics Committee at the Makerere University College of Health Sciences, and from the Uganda National Council for Science and Technology.

Methods

This study is registered in the Open Science Framework registries network (www.osf.io/registries) (DOI: 10.17605/OSF.IO/CN4M7). When preparing the protocol, we referred to relevant sections of the Cochrane Effective Practice and Organisation of Care (EPOC) Protocol and Review Template for Qualitative Evidence Synthesis (62).

Reflexivity

Reflexivity (63) is particularly important in this study, because: there is overlap between the teams responsible for the development of the intervention, the trials, the process evaluations, and this study; and the analysis will include the study team's experiences and views (see "Discussion").

We will apply a variety of individual and team-reflexive tools, including:

- reflexive notes kept by the primary investigators (PIs) in the process evaluations,
- a reflexive interview of the PIs in the process evaluations;
- team-reflexive discussions;
- a team-reflexive statement agreed on by the process evaluation teams;
- reflexive text where relevant in this protocol and the report of this study; and
- a narrative autobiography written by the PI in this study (MO) in parallel with the planning and conduct of the study.

As will become clear in the sections describing the analysis and synthesis, four members of the project team are particularly in position to influence this study: the PIs in the process evaluations, and MO, the primary investigator in this study. MO's subjectivity is summarised in

Box 1.

Box 1. MO's subjectivity.

For his doctoral project, MO has focused on potential adverse effects of the IHC secondary school intervention. Also, he removed himself from the development of the intervention due to unresolved disagreements about what were realistic effects, and he is concerned about systemic problems in research and education that might limit the intervention's benefits. For these reasons, he might exaggerate reports or observations of potential adverse effects. Moreover, MO is an outsider in the study contexts—a white, Western researcher in East-African secondary schools—which could cause misunderstandings. Finally, this study is part of MO's doctoral project, with limited financing, which could cause him to rush the study, limiting its rigour. In his narrative autobiography, MO is applying personal reflexivity to his subjectivity in more detail.

Search methods and selection criteria

Unlike a typical QES (59), this study excludes searching for and screening primary studies. There have been no other evaluations of the IHC secondary school intervention, so a search for such studies would be meaningless.

Data extraction

Descriptive data about included studies

MO will extract the following descriptive data verbatim from the reports of each process evaluation:

- study objectives, setting, and populations, and
- sampling and recruitment strategies.

Empirical data for the analysis and synthesis

The process evaluations include three sources of data:

- training and lesson evaluations forms filled in by teachers;
- classroom observation during the trials; and
- individual and group interviews after the trials, with students and teachers in the intervention arms, and other stakeholders, including parents and policymakers.

For this study, the PIs in the process evaluations will extract subsets of anonymised data and share them with MO (see "Indexing"). The subsets will include data about experiences and views of potential adverse effects in any individual or group, for example in parents, not just in recipients of the intervention (students and teachers).

Table 2 is an overview of the types of empirical data that we will include in the analysis and synthesis, with hypothetical examples.

Table 2. Types of empirical data to be included in the analysis and synthesis.

Experience/View	Data type		Hypothetical example
Participant	*Explicit participant reports*	Experiences views of specific, adverse outcomes, or lack thereof	• "I felt stressed while teaching the lessons."<br>• "I never felt stress because of the lessons"
		Views on specific, potential adverse effects of the IHC secondary school intervention	• "I think these lessons will cause teachers to feel stress."<br>• "I do not think the lessons can cause teachers to feel stress."
		General experiences or views of adverse effects of the IHC secondary school intervention	• "The lessons did not have any [adverse effects]."
		General experiences or views of adverse effects of educational interventions	• "I do not think education can have any [adverse effects]."
Study team	*Investigator observations*	Observations of outcomes that seem adverse	• A student said they would do their own study, to find the effects of the medicine.
	Implicit participant reports	Experiences or views of specific, adverse outcomes	• "I learned that I can do my own study, to find the effects of medicines."

In terms of data about participants' experiences and views, these will include explicit participant reports of:

- experiences or views of specific adverse outcomes, or lack thereof;
- views on specific, potential adverse effects of the IHC secondary school intervention;
- views on potential adverse effects of the IHC secondary school intervention in general; and
- views on potential adverse effects of educational interventions in general.

We will include data whether the participant uses the term "adverse [outcome or effect]" or plain-language alternatives such as "disadvantage", "downside", "harm", "negative", "bad", or "undesirable".

In terms of data about the study team's experiences and views, these will include:

- investigator observations of outcomes that seem adverse, and
- implicit participant reports of specific, adverse outcomes, such as misunderstandings.

Based on experiences developing the intervention, one such misunderstanding might be students thinking they have learned to do research and are able to find the effects of interventions themselves, as opposed to learning how to assess and use research evidence to inform decisions (see "Discussion").

MO will combine the empirical data from each trial setting in a single spreadsheet. With each data extract (quote or observation), he will include the trial setting (Kenyan, Rwandan, or Ugandan), the source (training or lesson evaluation form, interview transcript, or observation notes), and—if the source is an interview—the type of participant (student, teacher, or other, e.g., parent).

Assessing methodological limitations of the included studies

To assess the methodological limitations of the included studies, we will recruit an independent researcher with experience making such assessments. The assessment will be based on a list of domains iteratively developed by the Cochrane EPOC group (

Box 2) (64). MO will review the assessments, then he and the independent assessor will resolve any disagreements. If there are disagreements that they are unable to resolve, we will recruit a second independent assessor to mediate. The report of this study will include the final assessments in a methodological limitations table.

Box 2. Domains to be included in the assessment of methodological limitations.

- Were the settings and context described adequately?
- Was the sampling strategy described, and was this appropriate?
- Was the data collection strategy described and justified?
- Was the data analysis described, and was this appropriate?
- Were the claims made/findings supported by sufficient evidence?
- Was there evidence of reflexivity?
- Did the study demonstrate sensitivity to ethical concerns?
- Any other concerns?

Data management

We will store all data online, using a Microsoft SharePoint space administered by *Norsk helsenett*, the national e-health services provider in Norway. MO will give each data extract a unique identifier (e.g., data_001).

Analysis and synthesis

We will merge and modify framework analysis as outlined and exemplified by Ritchie and Spencer (65); framework synthesis as described by Barnett-Page *et*

al. (66), and exemplified by Brunton *et al.* (67) and Oliver *et al.* (68); and "best fit" framework synthesis as outlined and exemplified by Carrol *et al.* (69). All three are pragmatic approaches with a deductive analysis using an *a priori* framework, followed by an inductive thematic analysis.

Table 3 is an overview of the seven overarching stages of the analysis and synthesis.

Table 3. Stages of analysis and synthesis.

Trial settings	Study	Stage	
Across	Development of *a priori* framework (60)	1	Development of framework based on<br>• internal discussions,<br>• relevant evidence and theory,<br>• survey of experts, and<br>• interviews with teachers
Separate	Process evaluations (56–58)[1]	2	Familiarisation with data (65)
		3	Indexing (65)<br>• Rough coding<br>• Fine coding
		4	Charting (65)
		5	Mapping and interpretation (65)
Across	Qualitative Evidence Synthesis (this study)[1]	6	Synthesis<br>• Charting (65)<br>• Mapping and interpretation (65)
		7	Revision of framework (69)

[1]The process evaluations and this study overlap.

We will not develop a model, or test the synthesis and model, which are the final steps of "best fit" framework synthesis according to Carrol *et al.* (69). A model of potential adverse effects of the IHC secondary school intervention should also be based on findings about (potential) beneficial effects, given possible relationships between beneficial and adverse effects. For example, if the intervention has a beneficial effect in academically advantaged students, but not in academically disadvantaged students, the intervention also has an adverse effect on equity (60).

Development of a priori framework

In a separate study, we developed an *a priori* framework of potential adverse effects of the IHC secondary school intervention, including potential mechanisms, to inform the development and evaluation of the intervention (60). We developed the framework iteratively, based on internal discussions, relevant evidence and theory, a survey of researchers and others with a variety of relevant expertise, and interviews with teachers in Kenya, Rwanda, and Uganda.

This approach was like the approaches taken by Brunton *et al.* (67), and Oliver et al. (68), in their framework syntheses, developing *a priori* frameworks based on team discussions and relevant literature.

The framework includes a series of tables, providing an overview of potential adverse effects of the IHC secondary school intervention, as well as descriptions of potential mechanisms of the effects specified in the framework. The tables include:

- categories of adverse outcomes, with definitions;
- outcomes within those categories, with definitions;
- sub-outcomes;
- potentially affected individuals and groups; and
- corresponding beneficial outcomes.

Familiarisation

The PIs in the process evaluations are responsible for familiarisation (65) with all data collected for those studies: forms, recordings, transcripts, and notes. MO will have access to all the anonymised data, but only familiarise himself with those about potential adverse effects—excerpts from forms and transcripts, and notes—as coded by the PIs in the process evaluations. It is infeasible for MO to familiarise himself with all the process evaluation data from each trial setting.[1]

Indexing

In this study, the indexing stage (65) will have two parts: rough and fine coding. In the rough coding stage, the PIs in the process evaluation and at least one of their co-investigators in each trial setting will independently tag all data about views and experiences of adverse effects using a single, overarching theme. The PIs and their co-investigators will harmonise their rough coding. If any PI and co-investigator are unable to resolve any disagreements, another co-investigator will mediate. Then, the PIs will share the anonymised data with MO.

In the fine coding stage, MO and the PIs in the process evaluations will each independently code at least a sample of the data using a coding scheme based on the *a priori* framework (

[1]**Reflexive note:** Particularly the subjectivity of the PIs in the process evaluations might influence the familiarisation. MO's subjectivity (

Box 1) might also influence this stage, but he will only be familiarising himself with data based on coding by the PIs in the process evaluations (see "Indexing").

Table 4). The scheme includes themes for different categories of adverse outcomes, and sub-themes for adverse outcomes within those categories. The adverse effects would be increases in any of the outcomes.

Table 4. Initial coding scheme.

Theme (category of adverse outcome)		Sub-theme (outcome)	
Decision-making harms	Behaviours and beliefs that might contribute to poor choices	*Misunderstanding*	Incorrect understanding of a concept or example explained in the intervention
		Misapplication	Incorrect or unnecessary application of a skill or knowledge learned from the intervention
Psychological harms	Uncomfortable thoughts and feelings	*Distrust*	Feeling that a person or organisation cannot be relied on
		Pessimism	Inclination to believe that the application of skills or knowledge learned from the intervention is impossible or useless
		Cognitive dissonance	Experience of inconsistent beliefs
		Stress	Mental or emotional strain from work or schoolwork
Equity harms	Inequities in the distribution or size of effects	*Benefit-based inequity*	Inequity due to the distribution or size of a beneficial effect of the intervention
		Harm-based inequity	Inequity due to the distribution or size of an adverse effect of the intervention
Group and social harms	Harmful interactions between individuals, groups, populations, and systems	*Conflict*	Unconstructive argument between two or more parties
Waste	Waste of time and resources	*Wasted time*	Use of time on the intervention that would be better spent on other activity
		Wasted resources	Use of resources on the intervention that would be better spent on other activity

The coders will be free to alter the initial coding scheme according to new themes and sub-themes emerging from the data. Where there are reports or observations of a specific outcome, they will code for whether it is an explicit report, observation, or implicit report.

In addition to coding, they will note the generic terms that participants use to report adverse outcomes and potential adverse effects (see "Empirical data for the analysis and synthesis"). Also, they will note possible differences in how participants generally conceptualise adverse effects of educational

interventions, compared to the study team, and possible differences in adverse effects across trial settings, for exploration in later research (see “Discussion”).

After the independent coding, MO and each process evaluation PI will harmonise their coding. If they are unable to resolve any disagreements, another member of the study team will mediate.[2]

Charting

In the charting stage (65), MO will sort the data by theme (category of adverse outcome), then sub-theme (adverse outcome). He will copy the data, paste it in a document, in order, and abstract it into findings at the sub-theme level. During abstraction, we will not mix explicit participant reports with investigator observations, or implicit participant reports.

In their outline and examples of framework analysis, Ritchie and Spencer include separate entries in each chart for different individuals or groups (65). Doing so is not sensible in this study due to the mix of data sources and types, and the large number of participants in the process evaluations. Instead, MO will include separate entries for each trial setting.

At least one other member of the study team will check MO’s charting against the indexed data. MO and second team member will resolve any disagreements. If they are unable to resolve any disagreements, a third team member will mediate.[3]

Mapping and interpretation

In the mapping and interpretation stage (65), MO will explore experiences and views of potential adverse effects, and potential mechanisms, contrasting conflicting findings. This will include potential relationships between adverse effects, and experiences and views of potential adverse effects that were included in the *a priori* framework but not reported (explicitly or implicitly) or observed.

---

[2]**Reflexive note:** Particularly the subjectivity of the PIs in the process evaluations might influence the indexing. MO’s subjectivity (

Box 1) might also influence this stage, but he will only be fine coding data already rough coded by the PIs in the process evaluations.
[3]**Reflexive note:** Particularly MO’s subjectivity (

Box 1) might influence the charting. Applying reflexivity at this stage will be particularly important given judgements about abstracting direct quotes.

For the reports of the process evaluations, MO will write high-level summaries of the findings at the trial setting level, with reference to this study. For the reports of this study, MO will write more detailed summaries, reporting findings at the sub-theme (adverse outcome) level.

Synthesis

The synthesis stage is where the process evaluations stop, in terms of evaluating potential adverse effects, and this study continues. Given that it will be a synthesis of anonymised data, as opposed to reported findings, this stage will be a second round of charting, mapping, and interpreting, but across trial settings.

MO will conduct the synthesis. The other members of the study team will check MO's synthesis. At least one other member of the study team will check MO's charting against the indexed data. MO and second team member will resolve any disagreements. If they are unable to resolve any disagreements, a third member will mediate.[4]

Revision of framework

MO will revise the *a priori* framework (60). This includes adjusting, adding, or removing categories of adverse outcomes, and specific adverse outcomes, and revising definitions. The other members of the study team will review the changes by MO, or lack thereof, checking them against the findings.

Assessing confidence in the synthesis findings

MO and a second member of the study team with experience assessing confidence in QES findings will independently assess confidence in a sample of findings. MO and the second team member will harmonise their assessments, before MO assesses confidence in the remaining findings. The second team member will review the assessments made by MO. If MO and the second team member are unable to resolve any disagreements, a third team member will mediate.

The assessors will apply the Grading of Recommendations Assessment, Development and Evaluation Confidence in Evidence from Reviews of Qualitative research (GRADE-CERQual) approach (70), using the interactive Summary of Qualitative Findings tool (71). GRADE-CERQual assessments

---

[4]Like in the previous stage (see "Mapping and interpretation"), particularly MO's subjectivity (

Box 1) might influence the synthesis, and it will be particularly important to apply reflexivity at this stage given judgements about abstracting direct quotes.

include four components: methodological limitations, coherence, adequacy of data, and relevance. MO and the second investigator will assess confidence one component at a time—as opposed to one finding at a time—to help make the assessments of different components consistent across findings. To report the final assessment, MO will produce a Qualitative Evidence Profile, and Summary of Qualitative Findings Tables.

Discussion

Strengths

The QES and the process evaluations (the primary studies included in the QES) overlapping and being part of the same project (the development and evaluation of the IHC secondary school intervention) has methodological implications that amount to both strengths and limitations.

First, in terms of strengths, we will have in-depth knowledge about the subjectivity of researchers in the included studies. This facilitates comprehensive reflexivity.

Second, in planning the process evaluations, we harmonised the objectives, facilitating synthesis. However, investigators in each setting still had autonomy to explore phenomena specifically for their study. In this way, this QES is like a prospective meta-analysis (72)

Third, this study will be based on data from the process evaluations, facilitating for more reliable analysis and synthesis than if it was only based on reported findings, like in a typical QES (59). In this way, this QES is like an individual patient data (IPD) meta-analysis (73,74).

As far as we are aware, this QES will be the first of its kind in terms of similarities to prospective and IPD meta-analyses, and the first empirical study of its size and rigour focusing on potential adverse effects of an educational intervention.

Limitations

Overlap between studies

The QES and process evaluations overlapping and being part of the same project also introduces risk of bias. Unfortunately, it is infeasible to do member checking (75) in this study. However, we will recruit an independent researcher to assess the methodological limitations of the process evaluations (see "Assessing methodological limitations of the included studies"). In general, we will be transparent, and apply reflexivity throughout the planning, conduct, and report of this study (see "Reflexivity").

Inclusion of study team's experiences and views

Another challenge for this study is that we are including the study team's experiences and views. This introduces a risk of misrepresenting or downplaying the experiences and views of participants—especially because adverse outcomes and effects can be complex and variable.

An effect might be adverse in the experience or view of one individual or group, but not another. Moreover, the same individual might experience or view an effect as adverse in one context, but not another, or their experience or view might vary over time.

Health professionals and patients views on the importance of health outcomes, including adverse outcomes, sometimes vary substantially, and health professionals sometimes misunderstand patients' priorities (76–82). Similarly, the experiences and views of participants might vary from those of the study team, and the study team might misunderstand students', teachers', and other stakeholders' priorities.

The objective of this study is not to prioritise outcomes, but explore experiences and views of potential adverse effects, although the results could help inform such a prioritisation. The study team's experiences and views do not outweigh those of participants. However, certain adverse outcomes are difficult or inherently impossible for participants to recognise. In other words, if we excluded the study team's experiences and views, we might overlook potential adverse effects of the intervention.

For example, a participant cannot recognise that they have misunderstood a concept or example (see "Empirical data for the analysis and synthesis"). Causing such misunderstandings is a potential adverse effect since it might lead or contribute to poor decisions (60).

Also, it might be difficult for participants to acknowledge an experience as adverse. For example, a teacher might explicitly report that they had insufficient time, training, and resources for delivering the lessons, but not acknowledge it as stressful because of how they want to be perceived by their peers or investigators. Our aim would not be to determine whether the experience was in fact adverse, but to explore the possibility.

Moreover, even if an outcome was not adverse in one participant's experience, the same outcome might understandably be adverse in someone else's experience. Using the same example, one teacher might not experience a lack of time, training, and resources as adverse, but another might understandably experience it as such.

As noted in the methods section, during abstraction, we will not mix explicit reports of adverse outcomes or potential adverse effects with implicit reports, or observations (see "Charting"). In general, we will be transparent, and apply reflexivity throughout the planning, conduct, and report of this study (see "Reflexivity").

Differences in conceptualization or across trial settings

Fundamentally, there might be differences in how participants generally conceptualise adverse effects of educational interventions, compared to the study team. For example, some participants might not view educational interventions as being able to have adverse effects, like in the hypothetical example in

Table 2. However, we have not actively collected data about participants' conceptualisation.

Also, there might be differences in adverse effects across trial settings, given contextual differences such as varying levels of English fluency, or access to technology (83–85). However, we have not identified any obvious hypotheses for subgroup analyses, and there might not be rich enough data to explore any such hypotheses.

Where potential differences in conceptualisation, or differences across trial settings emerge, we will note them, for exploration in later research (see "Indexing").

Limited expertise

There are many fields of research, and lines of study within those fields, that are relevant to this QES and the project in general. In the project, we have tried to address this challenge by forming an interdisciplinary team and establishing an international advisory network with an even wider variety of expertise. However, the combined expertise of the team and network is not exhaustive. For example, there is limited expertise about the field of psychology, and the study of stress, an outcome included in the *a priori* framework (60). We welcome feedback from researchers in all relevant fields, and all stakeholders. The revised framework resulting from this study will be a "living" tool, which can be revised again and adapted for different purposes.

Adverse effects of "education as usual"

Finally, we have not explored experiences or views of potential adverse effects in the control arms of the trials. In education, "treatment as usual" or "standard care" might also have adverse effects.

Not learning how to think critically about health information and decisions might lead or contribute to poor decisions. In general, schools might already be exacerbating inequities between advantaged and disadvantaged children and young people.

In the report of this study, we will discuss the potential adverse effects of "education as usual" or "standard education", compared to the IHC secondary school intervention.

Funding, competing interests, data availability, and contributions to the protocol

Funding

The study is funded by the Research Council of Norway, as part of the project "Enabling sustainable public engagement in improving health and health equity", project number 284683, grant number 69006. The funder had no role in in the preparation or publication of this protocol.

Competing interests

The teaching resources we have developed in this project are freely available, and we do not stand to make any direct financial gain from people accessing or using them. However, it might be easier to publish reports of studies within the project, particularly in high-impact journals, and get funding to build on the project, if the findings suggest the resources and intervention are helpful and harmless. We will address this competing interest using team-reflexive tools (see "Reflexivity").

Data availability

For each process evaluation, we will anonymise and make available all included data (56–58). We will make the collated, anonymised data used in this study available via the open-access repository Zenodo (https://zenodo.org).

Peer review

We recruited Claire Glenton at the University of Western Norway, previously at NIPH, and Heather Ames at NIPH, to review the penultimate version of the protocol. CG and HA had not otherwise been involved in the development and evaluation of the IHC secondary school intervention. However, CG was involved in the development and evaluation of the IHC primary school intervention (5,38,86), on which the secondary schools project builds. Both CG and HA have worked with authors of the protocol on other projects.

Author contributions

MO and SL conceptualised the study. All authors reviewed at least one version of the protocol. Monica Melby-Lervåg at the Centre for Research on Special Needs Education and Inclusion, University of Oslo, provided early feedback on draft objectives, and is MO's co-supervisor. MO drafted and revised all versions of the protocol, published and registered the protocol, and organised the peer review.

References

1. Moberg J, Austvoll-Dahlgren A, Treweek S, Badenoch D, Layfield R, Harbour R, et al. The plain language Glossary of Evaluation Terms for Informed Treatment choices (GET-IT) at www.getitglossary.org. Research for All. 2018;2(1):106–21.
2. DECIDE, Informed Health Choices, Testing Treatments Interactive. GET-IT Glossary [Internet]. [cited 2021 Feb 4]. Available from: http://getitglossary.org
3. Oxman AD, Chalmers I, Dahlgren A. Key concepts for informed health choices. 2.1: Comparisons of treatments should be fair. J R Soc Med [Internet]. 2022 Dec 1;01410768221140733. Available from: https://doi.org/10.1177/01410768221140733
4. Booth A, Noyes J, Flemming K, Moore G, Tunçalp Ö, Shakibazadeh E. Formulating questions to explore complex interventions within qualitative evidence synthesis. BMJ Glob Health [Internet]. 2019 Jan 1;4(Suppl 1):e001107. Available from: http://gh.bmj.com/content/4/Suppl_1/e001107.abstract
5. Nsangi A, Semakula D, Glenton C, Lewin S, Oxman AD, Oxman M, et al. Informed health choices intervention to teach primary school children in low-income countries to assess claims about treatment effects: Process evaluation. BMJ Open. 2019;9(9):e030787.
6. Semakula D, Nsangi A, Oxman A, Glenton C, Lewin S, Rosenbaum S, et al. Informed Health Choices media intervention for improving people's ability to critically appraise the trustworthiness of claims about treatment effects: A mixed-methods process evaluation of a randomised trial in Uganda. BMJ Open. 2019;9(12).
7. Skivington K, Matthews L, Simpson SA, Craig P, Baird J, Blazeby JM, et al. A new framework for developing and evaluating complex interventions: update of Medical Research Council guidance. BMJ [Internet]. 2021;374. Available from: https://www.bmj.com/content/374/bmj.n2061
8. Zhao Y. What works may hurt: side effects in education. New York: Teachers College Press; 2018.
9. Zhao Y. What works may hurt: Side effects in education. Journal of Educational Change. 2017;18(1):1–19.

10. Smith SW, Hauben M, Aronson JK. Paradoxical and Bidirectional Drug Effects. Drug Saf [Internet]. 2012;35(3):173–89. Available from: https://doi.org/10.2165/11597710-000000000-00000
11. Bonell C, Jamal F, Melendez-Torres GJ, Cummins S. 'Dark logic': Theorising the harmful consequences of public health interventions. J Epidemiol Community Health (1978). 2015;69(1):95–8.
12. Humphrey N, Squires G, Choudry S, Byrne E, Demkowicz O, Troncos P, et al. Achievement for All: Evaluation Report [Internet]. 2020. Available from: https://educationendowmentfoundation.org.uk/public/files/Projects/Evaluation_Reports/Achievement_for_All_(final).pdf
13. Gamse BC, Bloom HS, Kemple JJ, Jacob RT. Reading First Impact Study: Interim Report. Washington, DC; 2008 Apr.
14. Cusack L, del Mar CB, Chalmers I, Hoffmann TC. Educational interventions to improve people's understanding of key concepts in assessing the effects of health interventions: a systematic review. Syst Rev. 2018;7(68).
15. Rowe MP, Marcus Gillespie B, Harris KR, Koether SD, Shannon LJY, Rose LA. Redesigning a general education science course to promote critical thinking. CBE Life Sci Educ. 2015;14(3):ar30.
16. Ouimet M, Lapointe L, Léon G. Examining the feasibility and impact of a graduate public administration course in evidence-informed policy. Evidence & Policy [Internet]. 2015;11(2):149–68. Available from: https://bristoluniversitypressdigital.com/view/journals/evp/11/2/article-p149.xml
17. Matic-Strametz M, Strametz R, Bohrt K, Ochsendorf F, Weberschock T. Students in Training to Become Biology Teachers - a controlled phase II trial (NCT01567267). Z Evid Fortbild Qual Gesundhwes. 2013;107(1):53–61.
18. Leshowitz B, Jenkens K, Heaton S, Bough TL. Fostering Critical Thinking Skills in Students with Learning Disabilities: An Instructional Program. J Learn Disabil. 1993;26(7):483–90.
19. Leshowitz B, DiCerbo KE, Okun MA. Effects of Instruction in Methodological Reasoning on Information Evaluation. Teaching of Psychology [Internet]. 2002 Jan 1;29(1):5–10. Available from: https://doi.org/10.1207/S15328023TOP2901_02
20. Kaelin MA, Huebner WW, Nicolich MJ, Kimbrough ML. Field Test of an Epidemiology Curriculum for Middle School Students. Am J Health Educ. 2007;38(1):16–31.
21. Derry SJ, Levin JR, Osana HP, Jones MS. Developing Middle-School Students' Statistical Reasoning Abilities Through Simulation

Gaming. In: Lajoie SP, editor. Reflections on Statistics. 1st ed. New York: Routledge; 1998. p. 175–95.
22. Ciarocco NJ, Lewandowski GW, van Volkom M. The Impact of a Multifaceted Approach to Teaching Research Methods on Students' Attitudes. Teaching of psychology. 2013;40(1):20–5.
23. Berger B, Steckelberg A, Meyer G, Kasper J, Mühlhauser I. Training of patient and consumer representatives in the basic competencies of evidence-based medicine: A feasibility study. BMC Med Educ. 2010;10(1):16.
24. Woloshin S, Schwartz LM, Welch HG. The Effectiveness of a Primer to Help People Understand Risk. Ann Intern Med [Internet]. 2007 Feb 20;146(4):256–65. Available from: https://www.acpjournals.org/doi/abs/10.7326/0003-4819-146-4-200702200-00004
25. Welch CE, van Lunen BL, Hankemeier DA. An evidence-based practice educational intervention for athletic trainers: A randomized controlled trial. J Athl Train. 2014;49(2):210–9.
26. Tait AR, Voepel-Lewis T, Levine R. Using digital multimedia to improve parents' and children's understanding of clinical trials. Arch Dis Child. 2015;100(6):589–93.
27. Ndebele PM, Wassenaar D, Munalula E, Masiye F. Improving understanding of clinical trial procedures among low literacy populations: an intervention within a microbicide trial in Malawi. BMC Med Ethics. 2012;13(1):29.
28. Kruse AY, Kjaergard LL, Krogsgaard K, Gluud C, Mortensen EL, Gottschau A, et al. A Randomized Trial Assessing the Impact of Written Information on Outpatients' Knowledge About and Attitude Toward Randomized Clinical Trials. Control Clin Trials. 2000;21(3):223–40.
29. Hendricks CC. Teaching Causal Reasoning Through Cognitive Apprenticeship: What Are Results From Situated Learning? The Journal of educational research (Washington, DC). 2001;94(5):302–11.
30. Dunn LB, Palmer BW, Keehan M. Understanding of placebo controls among older people with schizophrenia. Schizophr Bull. 2006;32(1):137–46.
31. Barnett K, Harrison C, Newman F, Bentley C, Cummins C. A randomised study of the impact of different styles of patient information leaflets for randomised controlled trials on children's understanding. Arch Dis Child. 2005;90(4):364–6.
32. Austvoll-Dahlgren A, Bjørndal A, Odgaard-Jensen J, Helseth S. Evaluation of a Web Portal for Improving Public Access to Evidence-Based Health Information and Health Literacy Skills: A

Pragmatic Trial. PLoS One [Internet]. 2012 May 31;7(5):e37715-. Available from: https://doi.org/10.1371/journal.pone.0037715
33. Steckelberg A, Hülfenhaus C, Kasper J, Mühlhauser I. Ebm@school – a curriculum of critical health literacy for secondary school students: results of a pilot study. Int J Public Health. 2009;54(3):158–65.
34. Santesso N, Rader T, Nilsen ES, Glenton C, Rosenbaum S, Ciapponi A, et al. A summary to communicate evidence from systematic reviews to the public improved understanding and accessibility of information: a randomized controlled trial. J Clin Epidemiol. 2015;68(2):182–90.
35. Semakula D, Nsangi A, Oxman AD, Oxman M, Austvoll-Dahlgren A, Rosenbaum S, et al. Effects of the Informed Health Choices podcast on the ability of parents of primary school children in Uganda to assess claims about treatment effects: a randomised controlled trial. The Lancet [Internet]. 2017 [cited 2017 May 29]; Available from: http://www.sciencedirect.com/science/article/pii/S0140673617312254
36. Nsangi A, Semakula D, Oxman AD, Austvoll-Dahlgren A, Oxman M, Rosenbaum S, et al. Effects of the Informed Health Choices primary school intervention on the ability of children in Uganda to assess the reliability of claims about treatment effects: a cluster-randomised controlled trial. The Lancet [Internet]. 2017;390(10092):374–88. Available from: http://www.sciencedirect.com.gate2.inist.fr/science/article/pii/S0140673617312266
37. Verdugo-Paiva F, Novillo F, Peña J, Ávila-Oliver C, Rada G. Screening (partial report). Update of: Educational interventions to improve people's understanding of key concepts in assessing the effects of health interventions. 2023 Jan 17 [cited 2023 Feb 9]; Available from: https://zenodo.org/record/7542970
38. Nsangi A, Semakula D, Oxman AD, Austvoll-Dahlgren A, Oxman M, Rosenbaum S, et al. Effects of the Informed Health Choices primary school intervention on the ability of children in Uganda to assess the reliability of claims about treatment effects, 1-year follow-up: A cluster-randomised trial. Trials. 2020;21(1).
39. Semakula D, Nsangi A, Oxman AD, Oxman M, Austvoll-Dahlgren A, Rosenbaum S, et al. Effects of the Informed Health Choices podcast on the ability of parents of primary school children in Uganda to assess the trustworthiness of claims about treatment effects: one-year follow up of a randomised trial. Trials [Internet].

2020;21(1):187. Available from: https://doi.org/10.1186/s13063-020-4093-x
40. Steckelberg A, Fuhlendorf A, Jonitz C, Mühlhauser I. ebm@school – a curriculum of critical health literacy for secondary school students: results of a qualitative study to explore possible adverse effects. Oslo; 2008.
41. Moher D, Hopewell S, Schulz KF, Montori V, Gøtzsche PC, Devereaux PJ, et al. CONSORT 2010 Explanation and Elaboration: updated guidelines for reporting parallel group randomised trials. BMJ [Internet]. 2010;340. Available from: https://www.bmj.com/content/340/bmj.c869
42. Ioannidis JPA, Evans SJW, Gøtzsche PC, O'Neill RT, Altman DG, Schulz K, et al. Better Reporting of Harms in Randomized Trials: An Extension of the CONSORT Statement. Ann Intern Med [Internet]. 2004 Nov 16;141(10):781–8. Available from: https://www.acpjournals.org/doi/abs/10.7326/0003-4819-141-10-200411160-00009
43. Lorenc T, Oliver K. Adverse effects of public health interventions: A conceptual framework. J Epidemiol Community Health (1978). 2014;68(3):288–90.
44. Oxman M, Larun L, Pérez Gaxiola G, Alsaid D, Qasim A, Rose CJ, et al. Quality of information in news media reports about the effects of health interventions: Systematic review and meta-analyses [version 2; peer review: 4 approved]. F1000Res. 2022;10(433).
45. Dahlgren A, Furuseth-Olsen K, Rose CJ, Oxman AD. The Norwegian public's ability to assess treatment claims: results of a cross-sectional study of critical health literacy [version 2; peer review: 1 approved, 1 approved with reservations]. F1000Res. 2021;9(179).
46. Boutron I, Haneef R, Yavchitz A, Baron G, Novack J, Oransky I, et al. Three randomized controlled trials evaluating the impact of 'spin' in health news stories reporting studies of pharmacologic treatments on patients'/caregivers' interpretation of treatment benefit. BMC Med [Internet]. 2019;17(1). Available from: https://bmcmedicine.biomedcentral.com/articles/10.1186/s12916-019-1330-9
47. Albarqouni L, Arab-Zozani M, Abukmail E, Greenwood H, Pathirana T, Clark J, et al. Overdiagnosis and overuse of diagnostic and screening tests in low-income and middle-income countries: a scoping review. Vol. 7, BMJ Global Health. 2022.
48. Albarqouni L, Palagama S, Chai J, Pathirana T, Bakhit M, Arab-zozani M, et al. Overuse of medications in low- and middle-income countries: a scoping review. 2022.

49. Brownlee S, Chalkidou K, Doust J, Elshaug AG, Glasziou P, Heath I, et al. Evidence for overuse of medical services around the world. The Lancet. 2017;390(10090):156–68.
50. Glasziou P, Straus S, Brownlee S, Trevena L, Dans L, Guyatt G, et al. Evidence for underuse of effective medical services around the world. The Lancet. 2017;390(10090):169–77.
51. Sharples JM, Oxman AD, Mahtani KR, Chalmers I, Oliver S, Collins K, et al. Critical thinking in healthcare and education. BMJ. 2017;357:j2234.
52. The Lancet. Why is health literacy failing so many? The Lancet. 2022 Nov 12;400(10364):1655.
53. Chesire F, Kaseje M, Ochieng M, Mugisha M, Ssenyonga R, Oxman M, et al. Effects of the Informed Health Choices secondary school intervention on the ability of lower secondary students in Kenya to think critically about health information and choices: Protocol for a cluster-randomized trial. 2022 May.
54. Mugisha M, Nyirazinyoye L, Simbi CMC, Chesire F, Ssenyonga R, Oxman M, et al. Effects of using the Informed Health Choices digital secondary school resources on the ability of Rwandan students to think critically about health: protocol for a cluster-randomised trial [Internet]. 2022 May [cited 2022 May 19]. Available from: https://zenodo.org/record/6562788
55. Ssenyonga R, Oxman AD, Nakyejwe E, Mugagga SK, Nsangi A, Semakula D, et al. Does the use of the Informed Health Choices teaching resources improve the secondary students' ability to critically think about health in Uganda? A cluster randomised trial protocol. 2022 May.
56. Chesire F, Kaseje M, Ochieng M, Mugisha M, Ssenyonga R, Oxman M, et al. Effect of the Informed Health Choices digital secondary school resources on the ability of lower secondary students in Kenya to critically appraise health claims: protocol for a process evaluation. 2022 Jul 28 [cited 2022 Oct 25]; Available from: https://zenodo.org/record/6919372
57. Mugisha M, Nyirazinyoye L, Oxman A, Simbi C, Chesire F, Ssenyonga R, et al. Use of the Informed Health Choices digital resources for teaching lower secondary school students in Rwanda to think critically about health: protocol for a process evaluation. 2022 Jul 21 [cited 2022 Oct 25]; Available from: https://zenodo.org/record/6874985
58. Ssenyonga R, Lewin S, Nakyejwe E, Nsangi A, Semakula D, Chesire F, et al. Informed heath choices intervention to teach secondary school adolescents in Uganda to assess claims about treatment

effects: A process evaluation protocol. 2022 Aug 12 [cited 2022 Oct 25]; Available from: https://zenodo.org/record/6984730
59. Noyes J, Booth A, Cargo M, Flemming K, Harden A, Harris J, et al. Chapter 21: Qualitative evidence. In: Higgins J, Thomas J, Chandler J, Cumpston M, Li T, Page M, et al., editors. Cochrane Handbook for Systematic Reviews of Interventions. version 6. Cochrane; 2022.
60. Oxman M, Chesire FC, Mugisha M, Ssenyonga R, Ngatia B, Nsangi A, et al. Potential adverse effects of an educational intervention: Development of a framework. medRxiv [Internet]. 2022 Jan 1;2022.07.27.22278097. Available from: http://medrxiv.org/content/early/2022/07/29/2022.07.27.22278097.abstract
61. Helse- og omsorgsdepartementet. Lov om medisinsk og helsefaglig forskning (helseforskningsloven) [Internet]. Lovdata. 2008 [cited 2022 Mar 21]. Available from: https://lovdata.no/dokument/NL/lov/2008-06-20-44
62. Glenton C, Bohren M, Downe S, Paulsen E, Lewin S, Effective Practice and Organisation of Care (EPOC). EPOC Qualitative Evidence Syntheses: Protocol and review template v1.3. 2022.
63. Olmos-Vega FM, Stalmeijer RE, Varpio L, Kahlke R. A practical guide to reflexivity in qualitative research: AMEE Guide No. 149. Med Teach. 2022;ahead-of-p(ahead-of-print):1–11.
64. Glenton C, Carlsen B, Lewin S, Wennekes MD, Winje BA, Eilers R, et al. Healthcare workers’ perceptions and experiences of communicating with people over 50 years of age about vaccination: a qualitative evidence synthesis. Cochrane Database of Systematic Reviews. 2021;2021(7).
65. Ritchie J, Spencer L. Qualitative data analysis for applied policy research. In: Analyzing qualitative data. 1994. p. 173–94.
66. Barnett-Page E, Thomas J. Methods for the synthesis of qualitative research: a critical review. BMC Med Res Methodol [Internet]. 2009;9(1):59. Available from: https://doi.org/10.1186/1471-2288-9-59
67. Brunton G, Oliver S, Oliver K, Lorenc T. A synthesis of research addressing children’s, young people’s and parents views of walking and cycling for transport. EPPI-Centre; 2006.
68. Oliver SR, Rees RW, Clarke-Jones L, Milne R, Oakley AR, Gabbay J, et al. A multidimensional conceptual framework for analysing public involvement in health services research. Health Expectations [Internet]. 2008 Mar 1;11(1):72–84. Available from: https://doi.org/10.1111/j.1369-7625.2007.00476.x
69. Carroll C, Booth A, Leaviss J, Rick J. ‘Best fit’ framework synthesis: refining the method. BMC Med Res Methodol. 2013;13(1):37.

70. Lewin S, Booth A, Glenton C, Munthe-Kaas H, Rashidian A, Wainwright M, et al. Applying GRADE-CERQual to qualitative evidence synthesis findings: introduction to the series. Implementation Science. 2018;13(1):2.
71. GRADE-CERQual Interactive Summary of Qualitative Findings (iSoQ). Oslo, Norway;
72. Seidler AL, Hunter KE, Cheyne S, Ghersi D, Berlin JA, Askie L. A guide to prospective meta-analysis. BMJ [Internet]. 2019;367. Available from: https://www.bmj.com/content/367/bmj.l5342
73. Stewart LA, Clarke MJ. Practical methodology of meta-analyses (overviews) using updated individual patient data. Stat Med [Internet]. 1995 Oct 15;14(19):2057–79. Available from: https://doi.org/10.1002/sim.4780141902
74. Stewart LA, Tierney JF. To IPD or not to IPD?: Advantages and Disadvantages of Systematic Reviews Using Individual Patient Data. Eval Health Prof [Internet]. 2002 Mar 1;25(1):76–97. Available from: https://doi.org/10.1177/0163278702025001006
75. Birt L, Scott S, Cavers D, Campbell C, Walter F. Member Checking: A Tool to Enhance Trustworthiness or Merely a Nod to Validation? Qual Health Res [Internet]. 2016 Jun 22;26(13):1802–11. Available from: https://doi.org/10.1177/1049732316654870
76. Coulman KD, Howes N, Hopkins J, Whale K, Chalmers K, Brookes S, et al. A Comparison of Health Professionals' and Patients' Views of the Importance of Outcomes of Bariatric Surgery. Obes Surg [Internet]. 2016;26(11):2738–46. Available from: https://doi.org/10.1007/s11695-016-2186-0
77. Festen S, Stegmann ME, Prins A, van Munster BC, van Leeuwen BL, Halmos GB, et al. How well do healthcare professionals know of the priorities of their older patients regarding treatment outcomes? Patient Educ Couns [Internet]. 2021;104(9):2358–63. Available from: https://www.sciencedirect.com/science/article/pii/S073839912100166X
78. Sathanapally H, Sidhu M, Fahami R, Gillies C, Kadam U, Davies MJ, et al. Priorities of patients with multimorbidity and of clinicians regarding treatment and health outcomes: a systematic mixed studies review. BMJ Open [Internet]. 2020 Feb 1;10(2):e033445. Available from: http://bmjopen.bmj.com/content/10/2/e033445.abstract
79. Ramer SJ, McCall NN, Robinson-Cohen C, Siew ED, Salat H, Bian A, et al. Health Outcome Priorities of Older Adults with Advanced CKD and Concordance with Their Nephrology Providers' Perceptions. Journal of the American Society of Nephrology [Internet].

2018;29(12). Available from: https://journals.lww.com/jasn/Fulltext/2018/12000/Health_Outcome_Priorities_of_Older_Adults_with.13.aspx
80. Steinsvik EAS, D FS, Karol A, Per F, Anders W, A DA. Do Perceptions of Adverse Events Differ Between Patients and Physicians? Findings From a Randomized, Controlled Trial of Radical Treatment for Prostate Cancer. Journal of Urology [Internet]. 2010 Aug 1;184(2):525–31. Available from: https://doi.org/10.1016/j.juro.2010.04.009
81. Hart RA, Cabalo A, Bess S, Akbarnia BA, Boachie-Adjei O, Burton D, et al. Comparison of Patient and Surgeon Perceptions of Adverse Events After Adult Spinal Deformity Surgery. Spine (Phila Pa 1976) [Internet]. 2013;38(9). Available from: https://journals.lww.com/spinejournal/Fulltext/2013/04200/Comparison_of_Patient_and_Surgeon_Perceptions_of.5.aspx
82. Alonso-Coello P, Montori VM, Díaz MG, Devereaux PJ, Mas G, Diez AI, et al. Values and preferences for oral antithrombotic therapy in patients with atrial fibrillation: physician and patient perspectives. Health Expectations [Internet]. 2015 Dec 1;18(6):2318–27. Available from: https://doi.org/10.1111/hex.12201
83. Ssenyonga R, Sewankambo NK, Mugagga SK, Nakyejwe E, Chesire F, Mugisha M, et al. Learning to think critically about health using digital technology in Ugandan lower secondary schools: A contextual analysis. PLoS One [Internet]. 2022 Feb 2;17(2):e0260367. Available from: https://doi.org/10.1371/journal.pone.0260367
84. Chesire F, Ochieng M, Mugisha M, Ssenyonga R, Oxman M, Nsangi A, et al. Contextualizing critical thinking about health using digital technology in secondary schools in Kenya: a qualitative analysis. Pilot Feasibility Stud [Internet]. 2022;8(1):227. Available from: https://doi.org/10.1186/s40814-022-01183-0
85. Mugisha M, Uwitonze AM, Chesire F, Senyonga R, Oxman M, Nsangi A, et al. Teaching critical thinking about health using digital technology in lower secondary schools in Rwanda: A qualitative context analysis. PLoS One [Internet]. 2021 Mar 22;16(3):e0248773. Available from: https://doi.org/10.1371/journal.pone.0248773
86. Nsangi A, Semakula D, Rosenbaum SE, Oxman AD, Oxman M, Morelli A, et al. Development of the informed health choices resources in four countries to teach primary school children to assess claims about treatment effects: A qualitative study employing a user-centred approach. Pilot Feasibility Stud

[Internet]. 2020;6(1):18. Available from: https://doi.org/10.1186/s40814-020-00565-6
