# Supplementary material for: Participants’ experiences of potential adverse effects of an intervention to improve critical thinking about health choices: a qualitative cross-trial process evaluation in Kenya, Rwanda and Uganda
Source: BMJ Open. 2025 Oct 21;15(10):e104236. doi: 10.1136/bmjopen-2025-104236 (PMC12548629; doi:10.1136/bmjopen-2025-104236)
Supplement: Supplementary file 3 [file bmjopen-2025-104236.reviewer_comments.pdf]

PEER REVIEW HISTORY

BMJ Open publishes all reviews undertaken for accepted manuscripts. Reviewers are asked to complete a checklist review form and are provided with free text boxes to elaborate on their assessment. These free text comments are reproduced below.

ARTICLE DETAILS

Title (Provisional)

PARTICIPANTS' EXPERIENCES OF POTENTIAL ADVERSE EFFECTS OF AN INTERVENTION TO IMPROVE CRITICAL THINKING ABOUT HEALTH CHOICES: A QUALITATIVE CROSS-TRIAL PROCESS EVALUATION IN KENYA, RWANDA, AND UGANDA.

Authors

Oxman, Matt; Chesire, Faith; Mugisha, Michael; Ssenyonga, Ronald; Nsangi, Allen; Oxman, Andrew; Fretheim, Atle; Rosenbaum, Sarah; Kaseje, Margaret; Sewankambo, Nelson; Melby-Lervåg, Monica; Lewin, Simon

---

VERSION 1 - REVIEW

**Reviewer**	**1**
**Name**	**Pranic, Shelly**
**Affiliation**	**University of Split School of Medicine, Public Health**
**Date**	**23-Aug-2025**
**COI**	**None**

Patients' perspectives are essential surrounding an intervention. This study used qualitative methods to

obtain data from teachers, students, and parents about perceived harms from the Informed Health

Choices secondary school intervention. Please find suggestions below according to manuscript section

to clarify the reporting of the methods, thank you.

Introduction

1. P. 2, lines 58-59 - Please provide the PROPERO number or link to the protocol for this study referred to here and lines 89-90 on p. 7.

Methods

Data collection and extraction

1. P. 10, line 150 - Was there an interview guide created before the study? Which authors created the structured, semi-structured, or other type of interview questions? Were the interviews in person, and who conducted the interviews? How long were each of the interviews intended to be?

Indexing

2. P. 15, lines 201-202 - Were there discussions between investigators about the changes to the initial coding scheme as well as what to exclude? Which of the authors participated in making the changes?

Results

1. P. 19, Table 3 – Please include as a study limitation that parental and teacher interviews were absent in two out of the three study countries. This seemed like a missed opportunity to collect different context specific perspectives.

**Reviewer**	**2**
**Name**	**Lühnen, Julia**
**Affiliation**	**Charité Universitätsmedizin Berlin, Institute of Clinical Nursing Science**
**Date**	**24-Aug-2025**
**COI**	**None**

Dear authors,

This is an important and very well-written paper.

Adverse events are rarely considered in educational interventions. While some adverse events may have to be accepted in order to promote critical thinking, such as initial dissonance or conflicts in the personal and professional context, the results can help to optimise interventions. Participants can be prepared for these effects, and the course can facilitate reflection on how to deal with them.

The methodological approach is appropriate and is presented comprehensively and transparently (e.g., consideration of three RCTs, further development of a preliminary framework and detailed presentation of individual analysis steps). A protocol was published in advance, and any changes were presented in a comprehensible manner.

Note: You use the term stakeholder, which is very common. However, a paper has been published recommending that the term be replaced, as it has colonial connotations. The authors suggest the term “interest-holder”. Perhaps you will consider replacing the term.

Akl EA et. al. “Interest-holders”: A new term to replace “stakeholders” in the context of health research and policy. Cochrane Ev Synth. 2024;2:e70007. https://doi.org/10.1002/cesm.70007

VERSION 1 - AUTHOR RESPONSE

Reviewer 1:

Introduction

1. P. 2, lines 58-59 - Please provide the PROPERO number or link to the protocol for this study referred to here and lines 89-90 on p. 7.

The systematic review was a partial update and a pragmatic, *ad hoc* extension of an earlier systematic review (PROSPERO no. CRD42016033103). The new review was not registered in PROSPERO and had no protocol—a limitation that we address in that manuscript, which we have also submitted to *BMJ Open* (bmjopen-2025-108268).

Methods

Data collection and extraction

P. 10, line 150 - Was there an interview guide created before the study? Which authors created the structured, semi-structured, or other type of interview questions? Were the interviews in person, and who conducted the interviews? How long were each of the interviews intended to be?

Regarding interview guides, please see our response to the editor(s). Authors of the protocols created the guides. Interviews were all in-person. Per the protocols, we expected interviews to last 1-1.5 hours. We have added all this information to the manuscript. The primary investigators in the country-level process evaluations and this study, as well as local research assistants conducted the interviews. We have made this explicit in the revised manuscript.

Indexing

P. 15, lines 201-202 - Were there discussions between investigators about the changes to the initial coding scheme as well as what to exclude? Which of the authors participated in making the changes?

MO made the changes. He discussed changes with AO. We have added this information to the manuscript.

Results

1. P. 19, Table 3 – Please include as a study limitation that parental and teacher interviews were absent in two out of the three study countries. This seemed like a missed opportunity to collect different context specific perspectives.

We conducted interviews with parents and teachers in all countries but not group interviews with teachers in Rwanda or Uganda, and not individual interviews with parents in Kenya or Rwanda. We have noted this in the main text in the revised manuscript.

Reviewer 2:

This is an important and very well-written paper.

Adverse events are rarely considered in educational interventions. While some adverse events may have to be accepted in order to promote critical thinking, such as initial dissonance or conflicts in the personal and professional context, the results can help to optimise interventions. Participants can be prepared for these effects, and the course can facilitate reflection on how to deal with them.

The methodological approach is appropriate and is presented comprehensively and transparently (e.g., consideration of three RCTs, further development of a preliminary framework and detailed presentation of individual analysis steps). A protocol was published in advance, and any changes were presented in a comprehensible manner.

Thank you for the eloquent and encouraging summary.

Note: You use the term stakeholder, which is very common. However, a paper has been published recommending that the term be replaced, as it has colonial connotations. The authors suggest the term "interest-holder". Perhaps you will consider replacing the term.

Akl EA et. al. "Interest-holders": A new term to replace "stakeholders" in the context of health research and policy. Cochrane Ev Synth. 2024;2:e70007. https://doi.org/10.1002/cesm.70007

We were unaware of the colonial connotations of "stakeholder". We have replaced it throughout the manuscript with "interest-holder", as suggested. We have also added a reference to the article by Akl. *et al*.

VERSION 2 - REVIEW

**Reviewer**	**1**
**Name**	**Pranic, Shelly**
**Affiliation**	**University of Split School of Medicine, Public Health**
**Date**	**16-Sep-2025**
**COI**	

I thank the authors for incorporating the reviewers' and editors' suggestions to improve the reporting of the study. Regarding the current version, I think there are a few minor aspects that can be added to clarify the time frame in which the study was conducted in the "Setting" section of the revised abstract.

Additionally, please add another detail about the in-person interviews in the Data collection and extraction section. Specifically, were the interview responses audio-recorded and then transcribed for later coding? Thanks!

VERSION 2 - AUTHOR RESPONSE

Comment: "Regarding the current version, I think there are a few minor aspects that can be added to clarify the time frame in which the study was conducted in the 'Setting' section of the revised abstract."

Response: We have added the years during which we collected data, in the relevant section of the abstract.

Comment: "Additionally, please add another detail about the in-person interviews in the Data collection and extraction section. Specifically, were the interview responses audio-recorded and then transcribed for later coding?"

Response: Yes. We have noted this in the relevant section.
